# Difference in reproductive mode rather than ploidy explains niche differentiation in sympatric sexual and apomictic populations of *Potentilla puberula*

**DOI:** 10.1002/ece3.4992

**Published:** 2019-03-05

**Authors:** Henar Alonso‐Marcos, Flavia Domizia Nardi, Susanne Scheffknecht, Andreas Tribsch, Karl Hülber, Christoph Dobeš

**Affiliations:** ^1^ Department of Forest Genetics Austrian Research Centre for Forests Vienna Austria; ^2^ Department of Conservation Biology, Vegetation Ecology and Landscape Ecology University of Vienna Vienna Austria; ^3^ Department of Biosciences University of Salzburg Salzburg Austria; ^4^ Institute of Botany University of Natural Resources and Life Sciences Vienna Austria

**Keywords:** apomixis, ecological differentiation, European Alps, polyploidy, reproductive mode, sympatry

## Abstract

Apomicts tend to have larger geographical distributional ranges and to occur in ecologically more extreme environments than their sexual progenitors. However, the expression of apomixis is typically linked to polyploidy. Thus, it is a priori not clear whether intrinsic effects related to the change in the reproductive mode or rather in the ploidy drive ecological differentiation. We used sympatric sexual and apomictic populations of *Potentilla puberula* to test for ecological differentiation. To distinguish the effects of reproductive mode and ploidy on the ecology of cytotypes, we compared the niches (a) of sexuals (tetraploids) and autopolyploid apomicts (penta‐, hepta‐, and octoploids) and (b) of the three apomictic cytotypes. We based comparisons on a ploidy screen of 238 populations along a latitudinal transect through the Eastern European Alps and associated bioclimatic, and soil and topographic data. Sexual tetraploids preferred primary habitats at drier, steeper, more south‐oriented slopes, while apomicts mostly occurred in human‐made habitats with higher water availability. Contrariwise, we found no or only marginal ecological differentiation among the apomictic higher ploids. Based on the pronounced ecological differences found between sexuals and apomicts, in addition to the lack of niche differentiation among cytotypes of the same reproductive mode, we conclude that reproductive mode rather than ploidy is the main driver of the observed differences. Moreover, we compared our system with others from the literature, to stress the importance of identifying alternative confounding effects (such as hybrid origin). Finally, we underline the relevance of studying ecological parthenogenesis in sympatry, to minimize the effects of differential migration abilities.

## 1 INTRODUCTION

Asexual plants tend to have larger geographical distribution ranges and to occur at higher latitudes and elevations compared to their sexual progenitors (Bierzychudek, 1985; van Dijk, 2003; Hörandl, 2006; Hörandl, Cosendai, & Temsch, 2008), a pattern known as “geographical parthenogenesis” (Vandel, 1928). Such a geographical parthenogenesis is often linked to ecological differentiation among sexual and asexual populations, a phenomenon defined as “ecological parthenogenesis” (Bell, 1982). Specifically, asexuals tend to (a) inhabit cooler or drier habitats and (b) have broader ecological ranges (e.g., in *Erigeron,* Noyes, Gerling, & Vandervoort, 2006; *Paspalum intermedium*, Karunarathne et al., 2018; *Ranunculus auricomus*, Paule, Dunkel, Schmidt, & Gregor, 2018), albeit examples showing opposite trends (e.g., asexuals inhabit more humid habitats in *Limonium*, Caperta et al., 2017, and *Ranunculus kuepferi*, Kirchheimer et al., 2016; Schinkel et al., 2016) indicate the idiosyncratic response of taxa.

Opposing hypotheses attempt to explain the differences in ecogeographical ranges of sexual and asexual populations. Following the General‐Purpose Genotype model (Lynch, 1984), selection would favor clones with broad ecological tolerance due to environmental heterogeneity over time (Lynch, 1984; Parker, Selander, Hudson, Lester, & Lester, 1977; Vrijenhoek & Parker, 2009) indicating asexuals as generalists having broader ecological tolerances and allowing the occupation of wider ranges. In contrast, following the Frozen Niche Variation model (Vrijenhoek, 1984), selection favors newly formed asexual clonal lineages with ecological niche optima at the margins of the ecological potential of the sexual parents (i.e., specialists), determining a shift in ecological preferences and ultimately promoting colonization of new geographical areas (Cosendai, Wagner, Ladinig, Rosche, & Hörandl, 2013; de Kovel & de Jong, 2000; Vrijenhoek & Parker, 2009).

Ecological differentiation of sexuals and apomicts (i.e., plants reproducing asexually via seeds; Asker & Jerling, 1992) is often studied in the context of geographical parthenogenesis (e.g., Karunarathne et al., 2018; Lo, Stefanović, & Dickinson, 2013; Noyes et al., 2006; Paule et al., 2018). However, differences in the geographical distribution may reflect different biogeographical histories or dispersal limitation rather than differences in ecological preferences (Brown, Stevens, & Kaufman, 1996; Sexton, McIntyre, Angert, & Rice, 2009). Shifts in ecological niches may therefore occur subsequently to colonization via adaptive processes to the new local environment, rather than arising as a direct effect of polyploidization or reproductive shift (Kirchheimer et al., 2016). Sympatry of sexuals with apomicts (sensu Rivas, 1964) thus offers the opportunity to unveil the immediate role of ecological differentiation of cytotypes on their distribution.

Despite rare exceptions (Böcher, 1951; Dobeš, Koch, & Sharbel, 2006; Siena, Sartor, Espinoza, Quarín, & Ortiz, 2008), apomicts are polyploid (Asker & Jerling, 1992; Carman, 1997; Comai, 2005; Dickinson, Lo, & Talent, 2007) and often form so‐called amphi‐apomictic systems with sexual diploids (e.g., Asker, 1970; Bayer, 1997; Böcher, 1951; Campbell & Wright, 1996; Hörandl & Gutermann, 1999). As a consequence, geographical and ecological parthenogenesis may depend on intrinsic effects of polyploidy (i.e., nucleotypic effects, Levin, 1983), rather than those of asexuality itself (Bierzychudek, 1985; Vandel, 1940). Polyploidization may thereby affect the geographical and ecological distribution of taxa either as a direct result of nucleotypic effects (e.g., changes in gene expression patterns) or by subsequent divergence in gene function (Adams & Wendel, 2005; Blanc & Wolfe, 2004). Thus, it may promote ecological differentiation of cytotypes (Soltis et al., 2004; Visger et al., 2016) and extend ecological (Fawcett, Maere, & Peer, 2009) and geographical ranges (Stebbins, 1985).

In this study, we aim to examine the role of ploidy and reproductive mode on ecological parthenogenesis. As our model system, we study sexual and apomictic sympatric populations of *Potentilla puberula*(Rosaceae), a species comprising five ploidy cytotypes well differentiated in their reproductive mode: Sexual tetraploids contrast autopolyploid apomicts with higher ploidy (penta‐ to octoploids). Based on a ploidy screen of 238 populations in the Eastern European Alps, and climatic variables, soil and topographic data, we (a) investigate the existence of ecological parthenogenesis; and we (b) test for ploidy level‐mediated ecological differentiation among the apomictic cytotypes. In case ecological parthenogenesis is followed by no or only marginal effects of ploidy, we explain the potential ecological differences among sexuals and apomicts by reproductive mode.

## 2 MATERIALS AND METHODS

### 2.1 Study species

**Figure 1:**
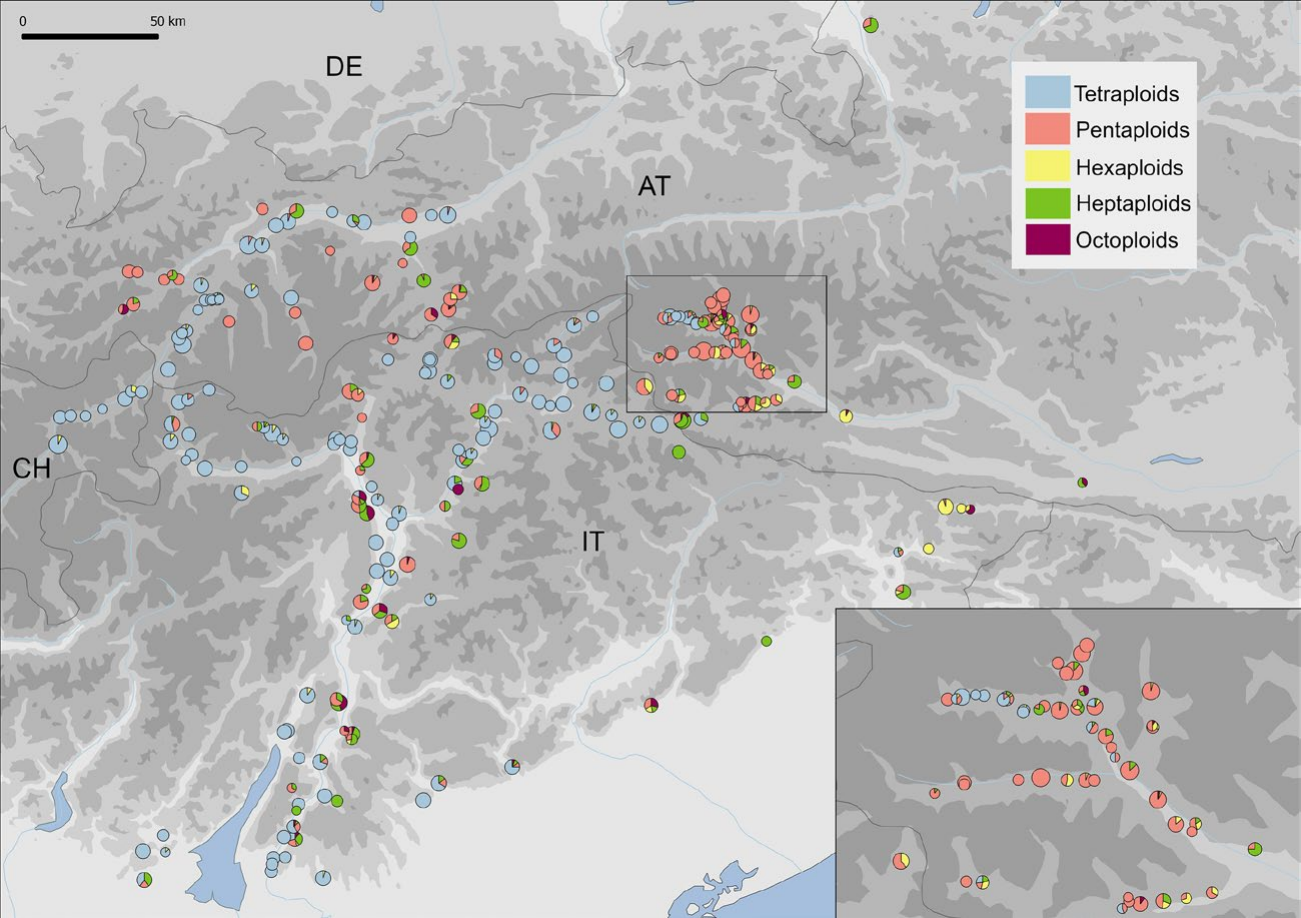
Cytotype composition at 238 populations (see Supporting Information Table A1 in Appendix S1) of *Potentilla puberula* in the Eastern European Alps. The pie charts represent the relative frequency of tetra‐, penta‐, hexa‐, hepta‐, and octoploids, and their size is proportional to the number of sampled individuals. AT, CH, DE, and IT: Austria, Switzerland, Germany, and Italy, respectively

The rosaceous species *Potentilla puberula*Krašan is an herbaceous plant growing preferably in xeric grasslands of the Eastern European Alps and Western Carpathians (Kurtto, Lampinen, & Junikka, 2004). It exhibits a series of reproductively differentiated ploidy levels comprising tetra‐ (*x* = 7; 2*n *= 28), penta‐ (2*n *= 35), hexa‐ (2*n *= 42), hepta‐ (2*n *= 49), and octoploids (2*n *= 56; Dobeš, 1999). Tetraploid individuals reproduce almost exclusively sexually and are self‐incompatible, whereas higher ploids are predominantly self‐compatible and pseudogamous apomicts (Dobeš et al., 2013, 2018; Nardi et al., 2018). For hexaploids, both reproductive modes are documented, with apomictic individuals usually clustering with other apomictic cytotypes, and sexual individuals occurring in low frequencies within sexual tetraploid populations (which probably originate frequently de novo as autohexaploids, Nardi et al., 2018). No fine‐scale cytogeography has ever been obtained across the whole distributional range (see Kurtto et al., 2004), although data collected by Dobeš (1999) suggest that only the apomictic cytotypes are present in Eastern Austria. On the contrary, sexual and apomictic cytotypes exhibit a sympatric distribution in the Eastern Alps (Figure 1; Dobeš et al., 2013; Nardi et al., 2018). Apomictic cytotypes are autopolyploid (i.e., being derived by duplication of a single genome; Nardi et al., 2018). Therefore, we could exclude potentially confounding effects of hybridization (Hörandl, 2006), as observed in allopolyploid model systems (e.g., in *Crataegus*, Coughlan, Stefanović, & Dickinson, 2014).

### 2.2 Study area and plant material

A total of 238 populations in the Eastern European Alps, between 45.53° and 47.78°N latitude and 10.10° and 13.81°E longitude, spanning an elevational range from 110 to 2,060 m a.s.l. were visited from 1999 to 2015 (Supporting Information Table A1 in Appendix S1). Per population, we collected an average of 15 individuals at least 2 m apart from each other and we kept them wet in zipped plastic bags for further cultivation in the experimental garden. We used Seradix B as a root‐inducing medium on the tips of the roots before potting and watering plants with the fungicide Previcur 0.15%. We drew a map of the study area with QGIS v.2.8.2 (QGIS Development Team, 2018).

### 2.3 DNA ploidy level estimation

We determined DNA ploidy levels (Suda, Krahulcová, Trávníček, & Krahulec, 2006), from now on referred to as ploidy levels, for convenience) of 3,716 individuals of *P. puberula* by flow cytometric analyses of fresh leaf petioles using the Partec Ploidy Analyser PA and Partec CyFlow (Partec, Münster, Germany) following the protocol of Doležel, Greilhuber, and Suda (2007). The ploidy of 1,838 individuals from 123 populations collected in 2015 was already presented and is described by Nardi et al. (2018). We estimated the ploidy of 1,878 additional individuals from 115 populations, collected during previous years (Supporting Information Table A1 in Appendix S1), using the standard two‐step (Otto) protocol described by Doležel et al., (2007) with an internal standard (*Solanum pseudocapsicum*: Temsch, Greilhuber, & Krisai, 2010; or *Lycopersicon esculentum* cv. Stupické polní tyčkové rané: Doležel & Bartoš, 2005). DAPI (4′‐6‐diamidino‐2‐phenylindole) served as DNA‐selective stain. We calculated the sample/standard fluorescence ratios from the means of the sample and standard fluorescence histograms. We considered only histograms with coefficients of variation (CVs) <5% for the *G*_0_/*G*_1_ peak of the analyzed sample. We then attributed ploidy levels based on the correlation of sample/standard fluorescence ratios with those of karyotyped individuals (Ptl4048, 2*n* = 4*x* = 28; Ptl4184, 2*n* = 5*x* = 35; Ptl4133, 2*n* = 7*x* = 49; Paule, Scherbantin, & Dobeš, 2012). In each measurement, we pooled individuals from the same population (usually five to eight).

### 2.4 Ecological variables

We combined climatic descriptors with topographic parameters, a variable representing the land use intensity and an index of vegetation density based on satellite imaginary. Elevation, inclination, and aspect (values of 0°, 180°, and 90° indicate facing direction north, south, and east/west, respectively) were used as topographic parameters. We assessed land use as primary (oligohemerobic, mostly natural grassland on shallow soils and rocky places) and secondary (mesohemerobic, i.e., anthropogenically disturbed pastures and meadows) habitats (Blume & Sukopp, 1976; Jalas, 1955) based on own field observations. We retrieved NDVI data (normalized difference vegetation index) from NASA through a Moderate Resolution Imaging Spectroradiometer (MODIS; modis.gsfc.nasa.gov) that incorporates a MOD13Q1 sensor (Didan, 2015), and we used it as index of vegetation density. Nineteen bioclimatic variables were retrieved unprocessed from the CHELSA Climate database (Karger et al., 2017a, 2017b; available at http://chelsa-climate.org/) with a spatial resolution of 30″. We performed Spearman correlation tests to identify highly correlated (|*r*| > 0.75) continuous variables (Supporting Information Table A2 in Appendix S1), and we reduced the set of variables to temperature seasonality (bio04), annual precipitation (bio12), precipitation seasonality (bio15), elevation, inclination, aspect, land use, and NDVI. In addition, we collected soil samples from 121 populations to analyze their pH, carbonate content, organic carbon and nitrogen content, and exchangeable cation content (see Supporting Information Appendix S2 and Table A3 in Appendix S1 for details). All obtained ecological data are available on Dryad (http://dx.doi.org/10.5061/dryad.bj0436h).

### 2.5 Statistical analyses

Given the strong correspondence between ploidy level and reproductive mode, with more than 90% of seeds by cytotype being derived by either reproductive mode (Dobeš et al., 2013, 2018; Nardi et al., 2018), we considered henceforth tetraploid individuals to be sexual and penta‐, hepta‐, and octoploids to be apomicts. According to Nardi et al. (2018), hexaploids comprise sexual autohexaploids (ca. 40% of genotypes) and apomicts (ca. 60%) and thus cannot be strictly associated with either sexual or apomictic reproductive mode. Therefore, we excluded hexaploid individuals from the analyses. To test for ecological differentiation, we performed pairwise comparisons of cytotypes separately for each ecological variable. Thereby, the probability of occurrence of a cytotype in a specific population (i.e., 0 or 1 in single‐cytotype populations and 0.5 in mixed populations) was related to ecological variables by means of logistic regressions. We used occurrences instead of frequencies (i.e., number of individuals) because of uneven sample sizes and to reduce the effects of possible reproductive interference among cytotypes (Dobeš et al., 2018), which may influence the relative frequencies of sexual and apomictic cytotypes in mixed populations. The Bonferroni correction was applied to the obtained *p*‐values. In addition, we compared sexual tetraploids to the pooled dataset of apomicts (i.e., penta‐, hepta‐, and octoploids) to test for ecological differences between reproductive modes. For all the analyses, we used the statistical computing environment R (R Development Core Team, 2018) and we represented the results by means of boxplots with the ggplot2 package (Wickham, 2016).

## 3 RESULTS

In total, we found 1,934 (52.0%) tetra‐, 1,080 (29.1%) penta‐, 196 (5.3%) hexa‐, 409 (11.0%) hepta‐, and 97 (2.6%) octoploid individuals in the 238 sampled populations (Figure 1). All cytotypes were present across the whole study area, but sexual tetraploids tended to occur in populations geographically separated from the apomictic ones (102, 74.45%), which were—in turn—usually formed by more than one cytotype (102, 76.12%; Figure 1). Individual flow cytometric measurements of the 115 populations not analyzed by Nardi et al. (2018) are available on Dryad (http://dx.doi.org/10.5061/dryad.bj0436h).

**Figure 2:**
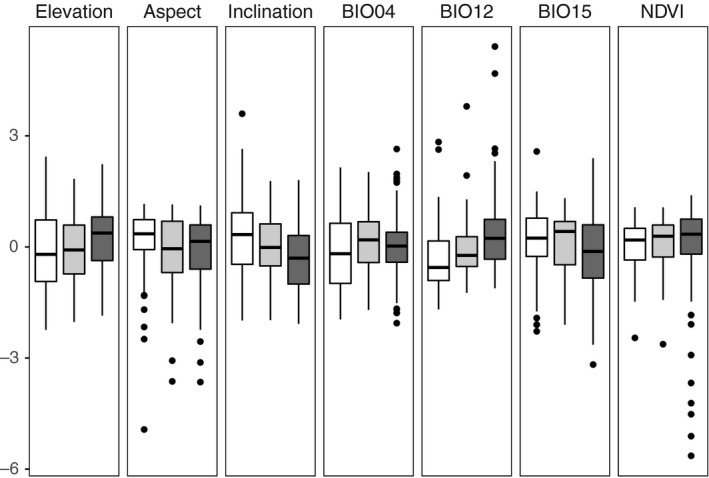
Boxplots of continuous ecological variables compared among 102 purely sexual (white), 99 purely apomictic (dark gray), and 35 mixed populations (light gray) of *Potentilla puberula*. Standardized values are reported. The boxes include the interquartile range (delimited by the 25th and the 75th percentiles), with the median reported as a horizontal line. The whiskers include values beyond the quartiles and up to 1.5 times the interquartile range above and below the upper and lower quartiles, respectively. The dots represent outlier values. Abbreviations: temperature seasonality (BIO04), annual precipitation (BIO12), precipitation seasonality (BIO15), normalized difference vegetation index (NDVI).

Several ecological variables differed significantly between sexual and apomictic cytotypes (Table 1). First, sexual tetraploids were more (although not exclusively) associated with primary oligohemerobic habitats than the pooled dataset of apomicts (Table 1): 38 (37.25%) pure sexual populations inhabited this particular environment versus 5 (5.05%) pure apomictic and 8 (22.86%) mixed sexual–apomictic populations. In addition, sexual tetraploids occurred at drier (712.69 ± 168.31 mm/year; bio12), at steeper (40.95 ± 17.63°), and more south‐oriented (148.79 ± 28.72°) slopes, compared to the pooled dataset of apomictic cytotypes inhabiting wetter (812.23 ± 203.00 mm/year; bio12), gentler (33.01 ± 15.87°) slopes (Table 1, Figure 2). This generally applied for all comparisons between tetraploids and the apomictic cytotypes taken singularly, although tetraploids and octoploids significantly differed only in annual precipitation (bio12) after we applied the Bonferroni correction (Table 1). In addition, heptaploids and pentaploids inhabited populations with stronger temperature seasonality (bio04) and higher elevations than sexuals, respectively, but the significance of these differences did not persist after the Bonferroni correction. No significant differences among cytotypes arose from any of the studied soil variables (Supporting Information Table A4 in Appendix S1).

**Table 1:** Pairwise comparison of ecological variables indicating site conditions among sexual and apomictic cytotypes of *Potentilla puberula*

Variable	Sexuals vs. Apomicts				Apomicts vs. Apomicts		
	Tetraploids vs. Apomicts	Tetra‐ vs. Pentaploids	Tetra‐ vs. Heptaploids	Tetra‐ vs. Octoploids	Penta‐ vs. Heptaploids	Penta‐ vs. Octoploids	Hepta‐ vs. Octoploids
Elevation	−0.24 ± 0.12	−0.29 ± 0.13*	0.01 ± 0.14	0.05 ± 0.20	0.32 ± 0.15*	0.35 ± 0.19	0.05 ± 0.21
Inclination	**0.50 ± 0.13** ***	**0.49 ± 0.14** ***	**0.56 ± 0.16** ***	0.59 ± 0.22**	0.08 ± 0.15	0.12 ± 0.20	0.04 ± 0.22
Aspect	**0.25 ± 0.12** *	0.24 ± 0.13	**0.39 ± 0.14** **	0.34 ± 0.17*	0.17 ± 0.14	0.16 ± 0.19	0.00 ± 0.21
Land use	**−1.55 ± 0.34** ***	**−1.59 ± 0.36** ***	**−1.40 ± 0.42** ***	−1.19 ± 0.57*	0.20 ± 0.49	0.40 ± 0.62	0.21 ± 0.66
NDVI	0.02 ± 0.12	−0.04 ± 0.13	−0.04 ± 0.15	−0.07 ± 0.21	0.00 ± 0.16	−0.02 ± 0.22	−0.01 ± 0.23
Bio04	−0.06 ± 0.12	0.02 ± 0.12	−0.30 ± 0.15*	−0.33 ± 0.20	−0.37 ± 0.16*	−0.39 ± 0.21	−0.05 ± 0.22
Bio12	**−0.62 ± 0.15** ***	**−0.59 ± 0.16** ***	**−0.64 ± 0.17** ***	**−0.74 ± 0.20** ***	−0.11 ± 0.14	−0.23 ± 0.20	−0.10 ± 0.22
Bio15	0.18 ± 0.12	0.15 ± 0.13	0.25 ± 0.15	0.37 ± 0.19	0.10 ± 0.14	0.24 ± 0.21	0.13 ± 0.22

Note

Coefficients of regression ± standard errors are given. Positive and negative coefficients indicate higher and lower values for the cytotypes given first in the column headings, respectively. Significance levels are indicated as: *p < 0.05; **p < 0.01; ***p < 0.001; significant comparisons after Bonferroni correction (adjusted *p* < 0.05) are formatted in bold.

bio04: temperature seasonality; bio12: annual precipitation; bio15: precipitation seasonality; NDVI: Normalized difference vegetation index.

Among the apomictic cytotypes, the regression analyses revealed no significant ecological differentiation. The exceptions of pentaploids occurring at populations at lower temperature seasonality (bio04) and at higher elevation compared to heptaploids did not show significant values after the adjustment of probability with the Bonferroni correction (Table 1).

## 4 DISCUSSION

We analyzed the ecological preferences of one sexual and three apomictic sympatric cytotypes of *P. puberula* in the Eastern European Alps. Tetraploids inhabited steeper and more south‐oriented slopes than the apomictic cytotypes, which, in turn, were associated with man‐made habitats with higher annual precipitation. The differences can be explained mainly by reproductive mode, since ecological occurrences were largely independent of the ploidy of the individuals, at least among the apomictic cytotypes.

### 4.1 Effects of reproductive mode versus ploidy and hybridity

Our results indicate that ecological differentiation among cytotypes of *P. puberula*is driven by differences in the reproductive mode rather than by effects of ploidy. Two lines of evidence support this argumentation: (a) The apomictic cytotypes hardly differed in their ecological niches; and (b) the niches of the apomictic cytotypes showed a strong differentiation from those of sexual tetraploids and these differences were similar for each of the three apomictic cytotypes.

Generally, niche differentiation among cytotypes can be expected due to the inherent differences in the number of genome copies which might cause differential gene expression through gene dosage effects or epigenetic changes (e.g., Baduel, Bray, Vallejo‐Marín, Kolář, & Yant, 2018; Comai, 2005; Parisod, Holderegger, & Brochmann, 2010; te Beest et al., 2012). However, studies on synthetic plant autopolyploids of several taxa indicate differences in expression patterns only for a minority of genes, suggesting that gene expression may change consistently only in allopolyploids (see Parisod et al., 2010 and references herein). In the autopolyploid *P. puberula,* indeed, our results suggest no nucleotypic effects. Since gene dosage effects depend by definition on the number of gene copies, we do not expect that differences in gene expression arise only between tetraploids and penta‐ to octoploids, but not among the apomictic cytotypes.

Since there is a strong link between ploidy level and reproductive mode (the low number of exceptional individuals prevents a stringent statistical test) and only one sexual cytotype exists, a priori our system does not allow a perfectly conclusive identification of the main driver of the observed ecological differentiation. However, it should be noted that (to our best knowledge), no perfect taxon is available to unambiguously separate the effects of reproductive mode, ploidy level, and in case of allopolyploidy, hybridity. Studies dealing with these issues often remained inconclusive, due to limitations inherent to the studied taxa, such as lack of cytotypic variation within reproductive mode or different evolutionary history of sexuals and apomicts (see Supporting Information Table A5 in Appendix S1 for a review of ecological studies in amphi‐apomictic systems). For instance, Schinkel et al., (2016) found apomictic individuals in three pure diploid populations of *Ranunculus kuepferi*, which shared the ecological site conditions of co‐occurring sexuals. However, their number was not high enough to prove that polyploidy was a requirement for range expansion. Kao, (2008) found no habitat differentiation between apomictic triploid and tetraploid *Arnica cordifolia*, similar to what we found in *P. puberula*within the apomictic cytotypes, but in her study, the comparison with sexual diploids was missing.

Perhaps the most complete attempt to address simultaneously the role of ploidy and reproductive mode on ecological differentiation of cytotypes is the genus‐wide study of Mau et al. (2015), who compared multiple species of *Boechera*, a taxon comprising both sexual and apomictic diploids and triploids. Oppositely to our findings in *P. puberula*, the authors concluded that ploidy, rather than mating system, determines a shift in ecological niches in this genus. Nevertheless, the results were not fully consistent, as niche differentiation among reproductive modes was observed at the diploid but not at the triploid level, and between ploidy levels in sexuals but not in apomicts (Mau et al., 2015). In contrast to *P. puberula*, hybridization plays an important role in the formation of apomicts in *Boechera* (e.g., Dobeš, Sharbel, & Koch, 2007) and a differential origin of apomictic cytotypes has been proposed: Apomictic diploids may originate intraspecifically while triploids derive by interspecific hybridization (Lovell et al., 2013).

Hybridization, often involved in the origin of apomixis (Grimanelli, Leblanc, Perotti, & Grossniklaus, 2001; Soltis & Soltis, 2009), may play an additional confounding role when studying the effects of reproductive mode on ecology. Genomic rearrangements, differential gene expression, and fixed heterozygosity are possible consequences of hybridization (Soltis & Soltis, 2000; te Beest et al., 2012; Wendel, 2000) leading to a shift of ecological niche of the progeny relative to those of the parents. Apomictic allopolyploids such as *Antennaria rosea* (Bayer, Purdy, & Lebedyk, 1991) and *Crataegus suksdorfii* and *Crataegus douglasii* (Coughlan, Han, Stefanović, & Dickinson, 2017) occupy ecological niches intermediate to those of the parental species, a hint that ecological niche shifts of those taxa indeed may be owing to their hybrid origin, rather than to apomixis itself. Contrariwise, in *Potentilla puberula*, the apomictic cytotypes are autopolyploid and genetically very similar to the sexual tetraploids (Nardi et al., 2018), ruling out strong effects of hybridization.

### 4.2 Evolutionary consequences of the ecological differentiation between sexuals and apomicts

Tetraploids occurred at drier, steeper, more south‐oriented, and oligohemerobic sites than apomicts. These primary habitats typically represented open forest (submediterranean deciduous forests and red pine forests in the southern and central parts of the Alps, respectively), natural xeric grasslands, and exposed rock vegetation. In contrast, apomicts preferred mesohemerobic habitats with a stronger anthropogenic impact such as mesic meadows and pastures of cattle, sheep, and goats on gentle slopes. The rarity of apomictic populations in primary habitats indicates that apomicts may benefit from human disturbance, establishing new populations and maintaining existing ones. Examples describing a stronger association of apomicts to anthropogenic habitat include *Taraxacum*sect. *Ruderalia* (Meirmans, Calame, Bretagnolle, Felber, & Nijs, 1999), *Ranunculus carpaticola* (Paun, Greilhuber, Temsch, & Hörandl, 2006), and Asiatic *Eupatorium*species (Watanabe, 1986). According to Eckert et al. (2010), anthropogenic disturbance has a disrupting effect on mating systems in plant populations, which can generate a shift toward increased rates of self‐fertilization in self‐compatible taxa (e.g., Luijten, Oostermeijer, Ellis‐Adam, & Nijs, 1999). Following this argumentation, self‐incompatible taxa such as tetraploid *P. puberula* might be easily outcompeted by self‐compatible (and apomictic) relatives. Moreover, low genetic diversity may be of advantage in moderately disturbed and unstructured habitats like meadows and pastures, where few clonal lineages may effectively exploit a homogeneous environment (Bell, 1982; Hörandl, 2006; Vrijenhoek, 1984). According to the Frozen Niche Variation hypothesis, clonal lineages can establish only when they occupy the margins of the sexuals’ niche (Vrijenhoek, 1984). Apomicts may thus establish preferentially in habitats where competition with sexuals is lower than in habitats at the sexuals’ niche optimum. Oligohemerobic and drier habitats, favored by sexual tetraploids, typically occur at the southern edge at the margin of the Alps toward the Po plain of our study area, which potentially has served as a glacial refugium for the montane to subalpine *P. puberula* (Tribsch & Schönswetter, 2003). The rather long‐term persistence of tetraploids at these southern populations, thus, would have promoted adaptation to local ecological conditions.

In fact, recombination is considered an advantage in habitats where either biotic (Red Queen hypothesis, Van Valen, 1973) or resource (Tangled Bank hypothesis, Bell, 1982; Song, Drossel, & Scheu, 2011) competition is high enough to promote high evolvability. Thus, the mating system may be associated with ecological traits related to the Competitive‐Stress‐tolerant‐Ruderal classification (Grime, 1974, 1977), in which asexuals mostly occur in marginal ruderal areas and tend to be less competitive than the outcrossers. Yang, Lascoux, and Glémin (2018) found evidence for this, testing for ecological strategies in four *Capsella* species, finding that selfers were more sensitive to competition than outcrossers.

A trend of apomictic plants to occur under more extreme environmental conditions compared to their sexual relatives has been repeatedly reported. Drought was identified as a factor driving the pattern in *Draba* (Price, 1980), *Antennaria* (Bierzychudek, 1985), *Bidens* (Crowe & Parker, 1981), *Paspalum intermedium* (Karunarathne et al., 2018), and the *Ranunculus auricomus* complex (Paule et al., 2018). The opposite pattern, however, was found in this study: Sexual *P. puberula*prevailed at drier sites, a tendency also observed in *Ranunculus kuepferi* (Schinkel et al., 2016). This might be explained by the negative effects of rainy weather on pollinators’ behavior (Puškadija et al., 2007), which avoid flying under the rain or foraging on wet blossoms. Frequent rain affects also flower display and can lead to closed inflorescences, as the case in dandelions (Mártonfiová, 2015). Therefore, sexual individuals, which depend on insect‐mediated outcross pollination, are likely to be favored in dry habitats in regions not affected by strong droughts, where water shortage become the dominant factor. Otherwise, in areas with higher precipitation, apomictic individuals can self‐fertilize independently of pollinators and (poor) weather conditions. Moreover, in moister habitats, fungal and microbial activity (Talley, Coley, & Kursar, 2002), as well as airborne spore abundance and release (see Crandall & Gilbert, 2017), is increased, which might indicate higher resistance of apomicts against pathogens. This would also explain why sexual individuals of *P*. *puberula*prefer steeper south‐exposed sites, which imply shallower and drier soils.

### 4.3 Ecological parthenogenesis in sympatry

The realized geographical range does not necessarily coincide with the potential range, which can be occupied by a taxon based only on its environmental requirements. Such range boundary disequilibria (Gaston, 2003; Sexton et al., 2009) might be due to dispersal limitation, colonization history, and even stochasticity (Sexton et al., 2009). In amphi‐apomictic systems, an advantage of apomicts in colonization given by uniparental reproduction (Baker's law; Baker, 1955; Stebbins, 1957) can be expected (Hao, Qiang, Chrobock, Kleunen, & Liu, 2011; Rambuda & Johnson, 2004). The breakdown of self‐incompatibility, common in both apomictic (Hörandl, 2010) and sexual polyploids (Barrett, 2002; Robertson, Goldberg, & Igić, 2011; but see Mable, 2004), provides the possibility of founding new populations and thus expanding the geographical range by a single individual even when pollination is still required (i.e., in pseudogamous apomicts, such as *P. puberula*, or in sexual selfers). Geographical range expansion of outcrossing sexuals, on the contrary, may be hindered by demographic dynamics (e.g., Allee effects, Allee, Emerson, Park, Park, & Schmidt, 1949) or pollinator dependence. Apomicts may thus have a priority effect (Levins & Culver, 1971; Shulman et al., 1983) in regions potentially suitable for sexuals and block their establishment by competitive exclusion and asymmetrical reproductive interference (Dobeš et al., 2018; Kyogoku, 2015; Mogie, 1992).

Given the central role that apomixis plays in colonization and range expansion, most studies on amphi‐apomictic systems focus on geographical parthenogenesis, rather than in purely ecological patterns (see Bierzychudek, 1985). Furthermore, in studies investigating the existence of ecological parthenogenesis, the geographical aspect is often neglected and conclusions on ecological differentiation between sexuals and apomicts are drawn without distinguishing between sympatric and allopatric populations (e.g., *Antennaria rosea*, Bayer et al., 1991; *Ranunculus carpaticola*, Paun et al., 2006; *Pilosella officinarum*, Mráz, Šingliarová, Urfus, & Krahulec, 2008; or *Paspalum intermedium*, Karunarathne et al., 2018; see Supporting Information Table A4 in Appendix S1). Ecological differentiation of allopatric apomicts may be in fact the result of postcolonization adaptation, or simply an artifact of range boundary disequilibria along ecogeographical gradients (Sexton et al., 2009).

While studying ecological differentiation in *Ranunculus kuepferi*, a species with clear geographical parthenogenesis, Kirchheimer et al. (2016) found that ecological niche shift and niche breadth were consistently larger in the allopatric apomictic populations, while the sympatric apomicts occupied a narrower niche at the margin of the sexual one. Therefore, the authors argued that the most relevant component of niche differentiation was derived by local adaptation which followed colonization of new areas (Kirchheimer et al., 2016).

In consequence, we affirm that sympatry of sexuals with apomicts is the most feasible scenario to separate the effects of immediate ecological differentiation from those arising as a consequence of range expansion. Nevertheless, only few studies used such sympatric systems or corrected for geographical distance (e.g., *Arnica*, Kao & Parker, 2010; *Boechera*, Mau et al., 2015; *Taraxacum*sect. *Ruderalia*, Meirmans et al., 1999; Verduijn, Dijk, & Damme, 2004; see Supporting Information Table A4 in Appendix S1). We studied ecological parthenogenesis of *P. puberula* in a transect through the Eastern Alps, where both sexuals and apomicts occur sympatrically in a mosaic‐like distribution (Figure 1). Given the strong association of apomicts with human‐disturbed habitats, one may argue that human dispersal may re‐introduce an effect of microcolonization patterns, even in a sympatric area. However, most sexual populations of *P. puberula* are found in mesohemerobic habitats as well. Therefore, we do not expect a preferential dispersal of apomicts due to human activity, and the effects of colonization patterns should be minimized in this sympatric region.

Associations between both reproductive mode and ploidy and ecological differentiation would need further experimental comparisons at multiple spatial scales with reciprocal transplant and common garden experiments, in which environmental parameters can be controlled for a clearer distinction between sexual and apomictic responses. Few studies tested for genetically fixed ecological differentiation in these terms, either between ploidy levels (e.g., Bretagnolle & Thompson, 2001; Münzbergová, 2007; Petit & Thompson, 1997) or reproductive modes (e.g., de Kovel & de Jong, 1999). When testing for differences between ploidy levels, the aforementioned authors did not find variation of phenotypic plasticity among cytotypes. The results were comparable to our lack of differences among the apomictic cytotypes of *P. puberula*. In *Taraxacum officinale*, apomictic triploids did not differ from sexual diploids in leaf height and length under lighted conditions, but they showed higher values under a shade treatment, suggesting that their phenotypic plasticity is higher than those of sexuals (de Kovel & de Jong, 1999). However, albeit the authors found significant differences in leaf plasticity between new and established triploids (de Kovel & de Jong, 2000), it is still unclear whether selection may have favored polyploidy or apomictic‐related traits.

In conclusion, we stress the importance of identifying and possibly neutralizing intermingled confounding factors (such as ploidy, hybrid origin, and spatial patterns) that could overestimate or mask the actual effects of reproductive mode in determining ecological parthenogenesis.

## CONFLICT OF INTEREST

The authors have no conflict of interest.

## AUTHOR CONTRIBUTIONS

CD, KH, FDN, and HAM designed the study. HAM, FDN, CD, SS, and AT carried out the field data collection. CD, HAM, and FDN performed the flow cytometric measurements. KH, FDN, and HAM performed the statistical analyses. HAM, FDN, KH, and CD wrote the manuscript. All authors critically commented on the results and the final version of the manuscript.

## Data Availability

All flow cytometric and ecological data acquired during this research and supporting this manuscript were deposited at the public repository Dryad (http://dx.doi.org/10.5061/dryad.bj0436h).
