# Supplementary material for: Difference in reproductive mode rather than ploidy explains niche differentiation in sympatric sexual and apomictic populations of Potentilla puberula
Source: Ecol Evol. 2019 Mar 5;9(6):3588–98. doi: 10.1002/ece3.4992 (PMC6434561; doi:10.1002/ece3.4992)
Supplement: Supplementary file 1 [file ECE3-9-3588-s001.docx]

**Appendix S1: Supplementary tables.**

**Table A1.** Description of 238 populations of *Potentilla puberula* sampled between 1999 and 2015 in the Eastern European Alps. Geographical and collection information, number of sampled individuals and cytotype composition variables, as well as source of flow cytometric data (FCM) and populations from which soil analyses were conducted (“x”) are given. Abbreviations: AT Andreas Tribsch; CD Christoph Dobeš; FDN Flavia Domizia Nardi; HAM Henar Alonso-Marcos; JAH Julian Ananda Haider; JP Juraj Paule; RS Roswitha Schmickl; SSc Susanne Scheffknecht; SSt Simon Stifter; TW Thomas Wilhalm.

| **Name** | **Longitude** | **Latitude** | **Country** | **Collection year** | **Collector** | **FCM** | **Soil subset** | **N** | **N 4*x*** | **N 5*x*** | **N 6*x*** | **N 7*x*** | **N 8*x*** | **N Sexuals** | **N Apomicts** |
| --- | --- | --- | --- | --- | --- | --- | --- | --- | --- | --- | --- | --- | --- | --- | --- |
| Affenhausen | 10.99608 | 47.30922 | Austria | 2015 | HAM, FDN | Nardi et al. (2018) | x | 18 | 0 | 6 | 0 | 12 | 0 | 0 | 18 |
| Ainet | 12.68615 | 46.86663 | Austria | 2009 | CD | present study |  | 9 | 0 | 9 | 0 | 0 | 0 | 0 | 9 |
| AlaE | 11.02516 | 45.7554 | Italy | 2015 | AT | Nardi et al. (2018) | x | 18 | 18 | 0 | 0 | 0 | 0 | 18 | 0 |
| Aldein | 11.35436 | 46.37103 | Italy | 2015 | HAM, FDN | Nardi et al. (2018) | x | 22 | 0 | 21 | 0 | 0 | 1 | 0 | 22 |
| Ambach | 10.86583 | 47.22028 | Austria | 2015 | HAM, FDN | Nardi et al. (2018) | x | 20 | 19 | 0 | 1 | 0 | 0 | 19 | 0 |
| Antholz | 12.099 | 46.84659 | Italy | 2011 | CD | present study |  | 20 | 20 | 0 | 0 | 0 | 0 | 20 | 0 |
| Arco Klettersteig | 10.88811 | 45.92728 | Italy | 2010 | CD | present study |  | 18 | 18 | 0 | 0 | 0 | 0 | 18 | 0 |
| Arco NE | 10.89694 | 45.93125 | Italy | 2015 | HAM, FDN | Nardi et al. (2018) | x | 19 | 19 | 0 | 0 | 0 | 0 | 19 | 0 |
| Ardez | 10.20403 | 46.77347 | Switzerland | 2015 | HAM, FDN | Nardi et al. (2018) | x | 10 | 10 | 0 | 0 | 0 | 0 | 10 | 0 |
| Arnig N | 12.6324 | 46.98423 | Austria | 2009 | CD | present study |  | 13 | 0 | 9 | 4 | 0 | 0 | 0 | 9 |
| Arzler Alm | 11.40576 | 47.29476 | Austria | 2015 | HAM, FDN | Nardi et al. (2018) | x | 20 | 0 | 20 | 0 | 0 | 0 | 0 | 20 |
| Auﬂer Klaunzerberg | 12.55929 | 46.97456 | Austria | 2009 | CD | present study |  | 20 | 0 | 16 | 0 | 4 | 0 | 0 | 20 |
| Avio | 10.93042 | 45.73367 | Italy | 2015 | AT | Nardi et al. (2018) | x | 14 | 14 | 0 | 0 | 0 | 0 | 14 | 0 |
| Avio 500 | 10.92178 | 45.71704 | Italy | 2013 | CD | present study |  | 5 | 0 | 0 | 0 | 5 | 0 | 0 | 5 |
| Bagni Lusnizza | 13.36923 | 46.5055 | Italy | 2015 | CD | Nardi et al. (2018) | x | 8 | 0 | 0 | 8 | 0 | 0 | 0 | 0 |
| Beseno | 11.10473 | 45.92957 | Italy | 2013 | CD | present study |  | 7 | 0 | 5 | 0 | 0 | 2 | 0 | 7 |
| Bichl | 12.539 | 46.98468 | Austria | 2015 | SSt, JAH | Nardi et al. (2018) | x | 12 | 5 | 6 | 0 | 1 | 0 | 5 | 7 |
| Birkach | 10.55203 | 46.97808 | Austria | 2010 | CD | present study |  | 18 | 16 | 0 | 2 | 0 | 0 | 16 | 0 |
| Bobojach | 12.40425 | 47.01677 | Austria | 2009 | CD | present study |  | 14 | 12 | 2 | 0 | 0 | 0 | 12 | 2 |
| Boedenalm | 11.69811 | 46.92309 | Italy | 2015 | HAM, FDN, SSt, JAH | Nardi et al. (2018) | x | 18 | 12 | 6 | 0 | 0 | 0 | 12 | 6 |
| Bozen Gries | 11.33083 | 46.50664 | Italy | 2010 | CD & SSc | present study |  | 20 | 19 | 0 | 1 | 0 | 0 | 19 | 0 |
| Brenito Belluno | 10.87395 | 45.64632 | Italy | 2015 | HAM, FDN, AT, CD | Nardi et al. (2018) | x | 16 | 16 | 0 | 0 | 0 | 0 | 16 | 0 |
| Bruneck | 11.9404 | 46.79348 | Italy | 2011 | CD | present study |  | 20 | 20 | 0 | 0 | 0 | 0 | 20 | 0 |
| Buchholz | 11.24806 | 46.24878 | Italy | 2015 | AT, FDN | Nardi et al. (2018) | x | 20 | 0 | 7 | 0 | 7 | 6 | 0 | 20 |
| Burg Berneck | 10.70447 | 47.07828 | Austria | 2010 | CD | present study |  | 8 | 8 | 0 | 0 | 0 | 0 | 8 | 0 |
| Burgfrieden | 12.71358 | 46.79813 | Austria | 2009 | CD | present study |  | 12 | 0 | 8 | 4 | 0 | 0 | 0 | 8 |
| Burgstall Kofler | 11.19472 | 46.62228 | Italy | 2006 | CD | present study |  | 8 | 0 | 6 | 0 | 2 | 0 | 0 | 8 |
| CampioloW | 13.13437 | 46.39156 | Italy | 2015 | CD | Nardi et al. (2018) | x | 7 | 4 | 2 | 0 | 1 | 0 | 4 | 3 |
| Canale | 10.83288 | 45.59015 | Italy | 2013 | CD | present study |  | 15 | 15 | 0 | 0 | 0 | 0 | 15 | 0 |
| Casalicolo | 10.44345 | 45.60779 | Italy | 2013 | CD | present study |  | 8 | 7 | 0 | 1 | 0 | 0 | 7 | 0 |
| Castelfeder | 11.29108 | 46.33633 | Italy | 2010 | CD & SSc | present study |  | 20 | 18 | 0 | 2 | 0 | 0 | 18 | 0 |
| Castellano | 11.43325 | 46.27797 | Italy | 2015 | AT, FDN | Nardi et al. (2018) | x | 12 | 11 | 0 | 1 | 0 | 0 | 11 | 0 |
| Ceraino | 10.82795 | 45.57418 | Italy | 2013 | CD | present study |  | 13 | 13 | 0 | 0 | 0 | 0 | 13 | 0 |
| Dabaklamm | 12.63125 | 47.02445 | Austria | 2009 | CD | present study |  | 27 | 0 | 26 | 1 | 0 | 0 | 0 | 26 |
| Eigenhofen | 11.19972 | 47.28222 | Austria | 2008 | CD | present study |  | 13 | 8 | 1 | 0 | 4 | 0 | 8 | 5 |
| Elvas | 11.66684 | 46.72881 | Italy | 2011 | CD | present study |  | 25 | 25 | 0 | 0 | 0 | 0 | 25 | 0 |
| Erlach Forststraﬂe | 12.3389 | 46.92442 | Austria | 2009 | CD | present study |  | 17 | 0 | 17 | 0 | 0 | 0 | 0 | 17 |
| Erlach Gehoeft | 12.33858 | 46.92237 | Austria | 2015 | SSt, JAH | Nardi et al. (2018) | x | 11 | 0 | 11 | 0 | 0 | 0 | 0 | 11 |
| Erlbach | 12.36958 | 46.74653 | Austria | 2009 | CD | present study |  | 25 | 0 | 6 | 0 | 15 | 4 | 0 | 25 |
| Eyrs | 10.63164 | 46.63105 | Italy | 2010 | CD & SSc | present study |  | 20 | 20 | 0 | 0 | 0 | 0 | 20 | 0 |
| Faggen1 | 10.67747 | 47.0765 | Austria | 2010 | CD | present study |  | 8 | 8 | 0 | 0 | 0 | 0 | 8 | 0 |
| Faggen2 | 10.67508 | 47.07758 | Austria | 2015 | HAM, FDN | Nardi et al. (2018) | x | 12 | 12 | 0 | 0 | 0 | 0 | 12 | 0 |
| Feld SSE | 12.57247 | 46.95087 | Austria | 2009 | CD | present study |  | 8 | 4 | 4 | 0 | 0 | 0 | 4 | 4 |
| Feldthurns | 11.60049 | 46.66362 | Italy | 2015 | CD | Nardi et al. (2018) | x | 12 | 11 | 0 | 1 | 0 | 0 | 11 | 0 |
| Fineil | 10.82583 | 46.74197 | Italy | 2015 | HAM, FDN | Nardi et al. (2018) | x | 8 | 0 | 4 | 0 | 4 | 0 | 0 | 8 |
| Finele | 11.16506 | 46.6992 | Italy | 2015 | HAM, FDN | Nardi et al. (2018) | x | 14 | 14 | 0 | 0 | 0 | 0 | 14 | 0 |
| Fischleinbach | 12.35239 | 46.66342 | Italy | 2011 | CD | present study |  | 15 | 0 | 0 | 0 | 15 | 0 | 0 | 15 |
| Fiss | 10.62539 | 47.05064 | Austria | 2010 | CD | present study |  | 9 | 9 | 0 | 0 | 0 | 0 | 9 | 0 |
| Flans | 11.46227 | 46.91411 | Italy | 2015 | CD | Nardi et al. (2018) | x | 12 | 12 | 0 | 0 | 0 | 0 | 12 | 0 |
| Flathalpe | 10.55844 | 47.13292 | Austria | 2015 | HAM, FDN | Nardi et al. (2018) | x | 11 | 0 | 11 | 0 | 0 | 0 | 0 | 11 |
| Flieﬂ | 10.63917 | 47.11611 | Austria | 2008 | CD | present study |  | 19 | 18 | 0 | 1 | 0 | 0 | 18 | 0 |
| Flirsch | 10.41042 | 47.15181 | Austria | 2015 | HAM, FDN | Nardi et al. (2018) | x | 11 | 0 | 11 | 0 | 0 | 0 | 0 | 11 |
| Forte Masua | 10.87572 | 45.59223 | Italy | 2015 | HAM, CD | Nardi et al. (2018) | x | 12 | 12 | 0 | 0 | 0 | 0 | 12 | 0 |
| Fosse | 10.9124 | 45.64068 | Italy | 2013 | CD | present study |  | 20 | 6 | 5 | 1 | 6 | 2 | 6 | 13 |
| Franzensfeste | 11.62985 | 46.77655 | Italy | 2015 | CD | Nardi et al. (2018) | x | 20 | 0 | 6 | 0 | 14 | 0 | 0 | 20 |
| Frosnitztal | 12.50262 | 47.04543 | Austria | 2009 | CD | present study |  | 17 | 0 | 17 | 0 | 0 | 0 | 0 | 17 |
| Gaid | 11.21383 | 46.50864 | Italy | 2010 | CD & SSc | present study |  | 20 | 0 | 0 | 0 | 11 | 9 | 0 | 20 |
| Gaium Croce | 10.82355 | 45.55506 | Italy | 2015 | HAM, FDN, AT, CD | Nardi et al. (2018) | x | 14 | 14 | 0 | 0 | 0 | 0 | 14 | 0 |
| Garniga Therme | 11.08814 | 46.00511 | Italy | 2011 | CD | present study |  | 24 | 0 | 1 | 6 | 6 | 11 | 0 | 18 |
| Garniga Vecchio | 11.08031 | 46.01447 | Italy | 2011 | CD | present study |  | 15 | 0 | 10 | 0 | 5 | 0 | 0 | 15 |
| Gemona | 13.14602 | 46.28551 | Italy | 2015 | CD | Nardi et al. (2018) | x | 19 | 0 | 4 | 2 | 13 | 0 | 0 | 17 |
| Gonzach | 12.66217 | 46.87545 | Austria | 2009 | CD | present study |  | 22 | 0 | 19 | 3 | 0 | 0 | 0 | 19 |
| Graun | 10.54503 | 46.80778 | Italy | 2010 | CD & SSc | present study |  | 20 | 20 | 0 | 0 | 0 | 0 | 20 | 0 |
| Greit | 10.56528 | 46.95917 | Austria | 2008 | CD | present study |  | 26 | 26 | 0 | 0 | 0 | 0 | 26 | 0 |
| Grezzana | 11.01033 | 45.5348 | Italy | 2015 | CD | Nardi et al. (2018) | x | 20 | 19 | 0 | 1 | 0 | 0 | 19 | 0 |
| Grins2 | 10.53644 | 47.14308 | Austria | 2010 | CD | present study |  | 9 | 0 | 3 | 0 | 6 | 0 | 0 | 9 |
| Grizzo | 12.64385 | 46.15783 | Italy | 2015 | CD | Nardi et al. (2018) | x | 9 | 0 | 0 | 0 | 9 | 0 | 0 | 9 |
| Groder | 12.33223 | 47.01812 | Austria | 2015 | SSt, JAH | Nardi et al. (2018) | x | 10 | 5 | 4 | 1 | 0 | 0 | 5 | 4 |
| Gruben | 12.51435 | 47.04818 | Austria | 2009 | CD | present study |  | 28 | 0 | 25 | 0 | 3 | 0 | 0 | 28 |
| Grumes | 11.29131 | 46.22039 | Italy | 2015 | AT, FDN | Nardi et al. (2018) | x | 18 | 0 | 6 | 9 | 3 | 0 | 0 | 9 |
| Guarda | 10.15072 | 46.77442 | Switzerland | 2010 | CD | present study |  | 12 | 12 | 0 | 0 | 0 | 0 | 12 | 0 |
| Guardia | 11.13076 | 45.90604 | Italy | 2013 | CD | present study |  | 11 | 0 | 3 | 2 | 6 | 0 | 0 | 9 |
| Hafling Oberdorf | 11.2195 | 46.65114 | Italy | 2015 | HAM, FDN | Nardi et al. (2018) | x | 19 | 0 | 7 | 0 | 11 | 1 | 0 | 19 |
| Heinfels Schloss | 12.43778 | 46.75024 | Austria | 2015 | SSt, JAH | Nardi et al. (2018) | x | 16 | 11 | 0 | 0 | 5 | 0 | 11 | 5 |
| Hinterbichl N | 12.34003 | 47.0202 | Austria | 2009 | CD | present study |  | 23 | 23 | 0 | 0 | 0 | 0 | 23 | 0 |
| Hochmuth | 11.12447 | 46.70395 | Italy | 2015 | HAM, FDN | Nardi et al. (2018) | x | 12 | 12 | 0 | 0 | 0 | 0 | 12 | 0 |
| Hof | 12.49742 | 46.92583 | Austria | 2009 | CD | present study |  | 13 | 0 | 6 | 7 | 0 | 0 | 0 | 6 |
| Hopfgarten | 12.52588 | 46.92562 | Austria | 2009 | CD | present study |  | 18 | 0 | 17 | 1 | 0 | 0 | 0 | 17 |
| Huben | 10.97861 | 47.04167 | Austria | 2015 | HAM, FDN | Nardi et al. (2018) | x | 12 | 0 | 12 | 0 | 0 | 0 | 0 | 12 |
| Innervillgraten | 12.36117 | 46.81235 | Austria | 2009 | CD | present study |  | 15 | 4 | 3 | 5 | 3 | 0 | 4 | 6 |
| Innichen | 12.28697 | 46.73611 | Italy | 2011 | CD | present study |  | 24 | 24 | 0 | 0 | 0 | 0 | 24 | 0 |
| Kaltern | 11.28192 | 46.38514 | Italy | 2010 | CD & SSc | present study |  | 18 | 18 | 0 | 0 | 0 | 0 | 18 | 0 |
| Katalalm | 12.49057 | 47.05703 | Austria | 2015 | SSt, JAH | Nardi et al. (2018) | x | 12 | 0 | 12 | 0 | 0 | 0 | 0 | 12 |
| Kauderle | 11.20222 | 46.30764 | Italy | 2010 | CD & SSc | present study |  | 7 | 0 | 2 | 0 | 5 | 0 | 0 | 7 |
| Kaunertal Maut | 10.73756 | 47.01928 | Austria | 2015 | HAM, FDN | Nardi et al. (2018) | x | 12 | 0 | 12 | 0 | 0 | 0 | 0 | 12 |
| Kauns | 10.69806 | 47.08028 | Austria | 2008 | CD | present study |  | 14 | 13 | 0 | 0 | 1 | 0 | 13 | 1 |
| Kematen | 11.54327 | 46.96069 | Italy | 2015 | CD | Nardi et al. (2018) | x | 19 | 0 | 8 | 6 | 3 | 2 | 0 | 13 |
| Kiens | 11.85225 | 46.79899 | Italy | 2011 | CD | present study |  | 18 | 18 | 0 | 0 | 0 | 0 | 18 | 0 |
| Kortsch | 10.76367 | 46.63458 | Italy | 2015 | HAM, FDN | Nardi et al. (2018) | x | 12 | 12 | 0 | 0 | 0 | 0 | 12 | 0 |
| Kosten | 12.60242 | 46.78637 | Austria | 2009 | CD | present study |  | 20 | 0 | 18 | 0 | 0 | 2 | 0 | 20 |
| Ladis | 10.65247 | 47.07605 | Austria | 2015 | HAM, FDN | Nardi et al. (2018) | x | 12 | 12 | 0 | 0 | 0 | 0 | 12 | 0 |
| Ladner | 10.39094 | 47.06797 | Austria | 2010 | CD | present study |  | 16 | 0 | 13 | 0 | 3 | 0 | 0 | 16 |
| Laengenfeld | 10.96583 | 47.08083 | Austria | 2015 | HAM, FDN | Nardi et al. (2018) | x | 20 | 20 | 0 | 0 | 0 | 0 | 20 | 0 |
| Lafairs | 10.565 | 46.99 | Austria | 2015 | HAM, FDN | Nardi et al. (2018) | x | 11 | 11 | 0 | 0 | 0 | 0 | 11 | 0 |
| Lago di Lago | 12.21845 | 45.98977 | Italy | 2015 | CD | Nardi et al. (2018) | x | 17 | 0 | 6 | 3 | 3 | 5 | 0 | 14 |
| Lana S | 12.63151 | 46.98602 | Austria | 2015 | SSt, JAH | Nardi et al. (2018) | x | 10 | 0 | 5 | 4 | 0 | 1 | 0 | 6 |
| Lasino | 10.97567 | 46.02592 | Italy | 2015 | AT, FDN | Nardi et al. (2018) | x | 21 | 19 | 0 | 2 | 0 | 0 | 19 | 0 |
| Latzfons | 11.55543 | 46.67389 | Italy | 2015 | CD | Nardi et al. (2018) | x | 12 | 12 | 0 | 0 | 0 | 0 | 12 | 0 |
| Lavin | 10.11092 | 46.77053 | Switzerland | 2010 | CD | present study |  | 15 | 15 | 0 | 0 | 0 | 0 | 15 | 0 |
| Luttach | 11.91475 | 46.94756 | Italy | 2015 | SSt, JAH | Nardi et al. (2018) | x | 19 | 16 | 3 | 0 | 0 | 0 | 16 | 3 |
| M Locherboden | 10.96294 | 47.28171 | Austria | 2011 | CD | present study |  | 21 | 20 | 1 | 0 | 0 | 0 | 20 | 1 |
| Maria Hilf | 12.29258 | 46.91262 | Austria | 2009 | CD | present study |  | 9 | 0 | 8 | 0 | 1 | 0 | 0 | 9 |
| Martell | 10.76136 | 46.5655 | Italy | 2011 | TW | present study |  | 19 | 13 | 0 | 6 | 0 | 0 | 13 | 0 |
| Matrei B | 11.45006 | 47.12288 | Austria | 2015 | HAM, FDN | Nardi et al. (2018) | x | 16 | 0 | 1 | 0 | 15 | 0 | 0 | 16 |
| Matrei NE | 12.54415 | 47.00783 | Austria | 2009 | CD | present study |  | 23 | 5 | 15 | 2 | 1 | 0 | 5 | 16 |
| Mattersberger | 12.56735 | 46.962 | Austria | 2009 | CD | present study |  | 10 | 0 | 10 | 0 | 0 | 0 | 0 | 10 |
| Mauls | 11.5227 | 46.85529 | Italy | 2011 | CD | present study |  | 18 | 16 | 0 | 0 | 2 | 0 | 16 | 2 |
| Maxer | 12.33606 | 46.81352 | Austria | 2009 | CD | present study |  | 11 | 0 | 11 | 0 | 0 | 0 | 0 | 11 |
| Melag | 10.65644 | 46.83958 | Italy | 2015 | HAM, FDN | Nardi et al. (2018) | x | 13 | 13 | 0 | 0 | 0 | 0 | 13 | 0 |
| Mellitz | 12.45663 | 46.9291 | Austria | 2009 | CD | present study |  | 29 | 0 | 29 | 0 | 0 | 0 | 0 | 29 |
| Mezzocorona | 11.12591 | 46.22478 | Italy | 2011 | CD | present study |  | 7 | 5 | 0 | 0 | 2 | 0 | 5 | 2 |
| Mezzomonte | 11.13336 | 45.92031 | Italy | 2015 | HAM, CD | Nardi et al. (2018) | x | 19 | 0 | 9 | 0 | 9 | 1 | 0 | 19 |
| Mieders | 11.37576 | 47.1694 | Austria | 2011 | CD | present study |  | 5 | 0 | 5 | 0 | 0 | 0 | 0 | 5 |
| Mignano | 11.70737 | 45.82835 | Italy | 2015 | CD | Nardi et al. (2018) | x | 20 | 15 | 2 | 0 | 2 | 1 | 15 | 5 |
| Monreale | 11.15578 | 46.20694 | Italy | 2015 | HAM, CD | Nardi et al. (2018) | x | 19 | 18 | 0 | 1 | 0 | 0 | 18 | 0 |
| Moos Passeier | 11.16847 | 46.83247 | Italy | 2015 | HAM, FDN | Nardi et al. (2018) | x | 22 | 0 | 16 | 0 | 6 | 0 | 0 | 22 |
| Mosson | 11.43974 | 45.78665 | Italy | 2015 | CD | Nardi et al. (2018) | x | 21 | 14 | 4 | 0 | 3 | 0 | 14 | 7 |
| Muehlbach | 11.97746 | 46.84848 | Italy | 2015 | SSt, JAH | Nardi et al. (2018) | x | 10 | 10 | 0 | 0 | 0 | 0 | 10 | 0 |
| Nals | 11.18589 | 46.53036 | Italy | 2015 | HAM, FDN | Nardi et al. (2018) | x | 19 | 0 | 10 | 0 | 6 | 3 | 0 | 19 |
| Nals Wehrburg | 11.18753 | 46.55028 | Italy | 2010 | CD & SSc | present study |  | 18 | 3 | 6 | 1 | 2 | 6 | 3 | 14 |
| Nauders | 10.51028 | 46.89417 | Austria | 2015 | HAM, FDN | Nardi et al. (2018) | x | 20 | 20 | 0 | 0 | 0 | 0 | 20 | 0 |
| Niedermauern | 12.43375 | 47.00242 | Austria | 2009 | CD | present study |  | 12 | 12 | 0 | 0 | 0 | 0 | 12 | 0 |
| Oberassling | 12.63775 | 46.78937 | Austria | 2015 | SSt, JAH | Nardi et al. (2018) | x | 19 | 0 | 9 | 4 | 6 | 0 | 0 | 15 |
| Oberbergtal | 11.26232 | 47.11823 | Austria | 2015 | HAM, FDN | Nardi et al. (2018) | x | 21 | 0 | 19 | 1 | 0 | 1 | 0 | 20 |
| Oberbichl | 12.36163 | 47.0224 | Austria | 2009 | CD | present study |  | 9 | 9 | 0 | 0 | 0 | 0 | 9 | 0 |
| Oberdrauburg | 12.96362 | 46.75371 | Austria | 2015 | CD | Nardi et al. (2018) | x | 15 | 0 | 0 | 14 | 0 | 1 | 0 | 1 |
| Oberegg | 13.112694 | 47.78117 | Austria | 2015 | AT, FDN | Nardi et al. (2018) |  | 20 | 0 | 6 | 0 | 14 | 0 | 0 | 20 |
| Obergaimberg | 12.7822 | 46.84623 | Austria | 2009 | CD | present study |  | 16 | 0 | 4 | 0 | 12 | 0 | 0 | 16 |
| Obermauern | 12.43528 | 47.00477 | Austria | 2015 | SSt, JAH | Nardi et al. (2018) | x | 12 | 11 | 0 | 1 | 0 | 0 | 11 | 0 |
| Oberpeischlach | 12.59413 | 46.93587 | Austria | 2009 | CD | present study |  | 30 | 0 | 26 | 0 | 4 | 0 | 0 | 30 |
| Obersteiner | 12.40872 | 47.01787 | Austria | 2015 | SSt, JAH | Nardi et al. (2018) | x | 18 | 11 | 4 | 1 | 2 | 0 | 11 | 6 |
| Olang | 12.04316 | 46.77096 | Italy | 2011 | CD | present study |  | 23 | 21 | 0 | 1 | 1 | 0 | 21 | 1 |
| Ossenigo | 10.90949 | 45.67443 | Italy | 2015 | HAM, FDN, AT, CD | Nardi et al. (2018) | x | 15 | 9 | 4 | 0 | 1 | 1 | 9 | 6 |
| Passeier Spath | 11.20694 | 46.76222 | Italy | 2010 | CD & SSc | present study |  | 6 | 0 | 6 | 0 | 0 | 0 | 0 | 6 |
| Passeier-Moos | 11.19567 | 46.82269 | Italy | 2015 | HAM, FDN | Nardi et al. (2018) | x | 14 | 0 | 12 | 2 | 0 | 0 | 0 | 12 |
| Patsch | 11.40512 | 47.20982 | Austria | 2015 | HAM, FDN | Nardi et al. (2018) | x | 20 | 0 | 7 | 0 | 13 | 0 | 0 | 20 |
| Pavone | 10.43776 | 45.65391 | Italy | 2015 | HAM, FDN, CD | Nardi et al. (2018) | x | 12 | 12 | 0 | 0 | 0 | 0 | 12 | 0 |
| Perdross | 10.57831 | 46.81395 | Italy | 2015 | HAM, FDN | Nardi et al. (2018) | x | 14 | 12 | 2 | 0 | 0 | 0 | 12 | 2 |
| Pettnau | 11.1253 | 47.30593 | Austria | 2015 | HAM, FDN | Nardi et al. (2018) | x | 11 | 11 | 0 | 0 | 0 | 0 | 11 | 0 |
| Pflersch Ende | 11.33019 | 46.96975 | Italy | 2015 | CD | Nardi et al. (2018) | x | 11 | 0 | 10 | 0 | 0 | 1 | 0 | 11 |
| Pfossertal Abzw | 10.91764 | 46.706 | Italy | 2015 | HAM, FDN | Nardi et al. (2018) | x | 12 | 11 | 0 | 1 | 0 | 0 | 11 | 0 |
| Pfunders | 11.69771 | 46.90367 | Italy | 2011 | CD | present study |  | 20 | 20 | 0 | 0 | 0 | 0 | 20 | 0 |
| Pians | 10.50642 | 47.13172 | Austria | 2015 | HAM, FDN | Nardi et al. (2018) | x | 11 | 0 | 11 | 0 | 0 | 0 | 0 | 11 |
| Planeil | 10.57711 | 46.72214 | Italy | 2010 | CD & SSc | present study |  | 17 | 17 | 0 | 0 | 0 | 0 | 17 | 0 |
| Plenten | 11.53583 | 47.04639 | Austria | 2015 | HAM, FDN | Nardi et al. (2018) | x | 20 | 0 | 17 | 0 | 1 | 2 | 0 | 20 |
| Pontebba | 13.31276 | 46.51041 | Italy | 2015 | CD | Nardi et al. (2018) | x | 21 | 0 | 1 | 20 | 0 | 0 | 0 | 1 |
| Praegraten N | 12.37452 | 47.02117 | Austria | 2015 | SSt, JAH | Nardi et al. (2018) | x | 12 | 12 | 0 | 0 | 0 | 0 | 12 | 0 |
| Praemajur | 10.50964 | 46.70414 | Italy | 2010 | CD & SSc | present study |  | 20 | 18 | 0 | 2 | 0 | 0 | 18 | 0 |
| Prags | 12.13593 | 46.72472 | Italy | 2011 | CD | present study |  | 25 | 25 | 0 | 0 | 0 | 0 | 25 | 0 |
| Punt de la Resgia | 10.40986 | 46.83493 | Switzerland | 2010 | CD | present study |  | 13 | 13 | 0 | 0 | 0 | 0 | 13 | 0 |
| Raas W | 11.6535 | 46.74708 | Italy | 2015 | CD | Nardi et al. (2018) | x | 12 | 11 | 0 | 1 | 0 | 0 | 11 | 0 |
| Rabenstein | 12.4664 | 47.00887 | Austria | 2015 | SSt, JAH | Nardi et al. (2018) | x | 12 | 0 | 12 | 0 | 0 | 0 | 0 | 12 |
| Ramosch | 10.3739 | 46.83735 | Switzerland | 2010 | CD | present study |  | 13 | 9 | 0 | 4 | 0 | 0 | 9 | 0 |
| Raneburg | 12.52795 | 47.0673 | Austria | 2009 | CD | present study |  | 25 | 0 | 25 | 0 | 0 | 0 | 0 | 25 |
| Ratzell | 12.53903 | 46.92542 | Austria | 2015 | SSt, JAH | Nardi et al. (2018) | x | 12 | 0 | 12 | 0 | 0 | 0 | 0 | 12 |
| Raut | 12.5748 | 46.78128 | Austria | 2015 | SSt, JAH | Nardi et al. (2018) | x | 9 | 5 | 4 | 0 | 0 | 0 | 5 | 4 |
| Reifenstein | 11.44342 | 46.87856 | Italy | 2015 | CD | Nardi et al. (2018) | x | 12 | 12 | 0 | 0 | 0 | 0 | 12 | 0 |
| Ridnaun | 11.3093 | 46.91546 | Italy | 2015 | CD | Nardi et al. (2018) | x | 12 | 12 | 0 | 0 | 0 | 0 | 12 | 0 |
| Rodenegg | 11.69085 | 46.77489 | Italy | 2011 | CD | present study |  | 16 | 16 | 0 | 0 | 0 | 0 | 16 | 0 |
| Ronchi | 11.06998 | 45.74151 | Italy | 2015 | HAM, FDN, CD | Nardi et al. (2018) | x | 12 | 0 | 0 | 0 | 12 | 0 | 0 | 12 |
| Roppen | 10.81667 | 47.22083 | Austria | 2008 | CD | present study |  | 28 | 26 | 2 | 0 | 0 | 0 | 26 | 2 |
| Rossbach | 10.87171 | 47.31492 | Austria | 2015 | HAM, FDN | Nardi et al. (2018) | x | 12 | 0 | 12 | 0 | 0 | 0 | 0 | 12 |
| Roveredo | 13.24515 | 46.39924 | Italy | 2015 | CD | Nardi et al. (2018) | x | 10 | 0 | 0 | 10 | 0 | 0 | 0 | 0 |
| S. Valentino | 10.907799 | 45.77776 | Italy | 2015 | AT, FDN | Nardi et al. (2018) |  | 6 | 0 | 4 | 0 | 2 | 0 | 0 | 6 |
| Saeben Burg | 11.56974 | 46.64646 | Italy | 2011 | CD | present study |  | 19 | 18 | 0 | 1 | 0 | 0 | 18 | 0 |
| Saeben N | 11.58138 | 46.65169 | Italy | 2015 | CD | Nardi et al. (2018) | x | 18 | 3 | 4 | 0 | 11 | 0 | 3 | 15 |
| San Eusebio | 10.36253 | 45.61144 | Italy | 2015 | HAM, FDN, CD | Nardi et al. (2018) | x | 20 | 20 | 0 | 0 | 0 | 0 | 20 | 0 |
| Santantonio | 10.93782 | 45.85773 | Italy | 2015 | HAM, CD | Nardi et al. (2018) | x | 12 | 12 | 0 | 0 | 0 | 0 | 12 | 0 |
| Santosso | 11.38215 | 45.74177 | Italy | 2015 | CD | Nardi et al. (2018) | x | 20 | 20 | 0 | 0 | 0 | 0 | 20 | 0 |
| Scaiola | 10.36401 | 45.53424 | Italy | 2015 | HAM, CD | Nardi et al. (2018) | x | 20 | 8 | 4 | 0 | 8 | 0 | 8 | 12 |
| Schlaneid | 11.23403 | 46.57875 | Italy | 2010 | CD & SSc | present study |  | 11 | 11 | 0 | 0 | 0 | 0 | 11 | 0 |
| Schluderns | 10.58537 | 46.66813 | Italy | 2015 | HAM, FDN | Nardi et al. (2018) | x | 14 | 14 | 0 | 0 | 0 | 0 | 14 | 0 |
| Schmirn | 11.54472 | 47.07333 | Austria | 2008 | CD | present study |  | 16 | 0 | 12 | 4 | 0 | 0 | 0 | 12 |
| Schmuders | 11.46227 | 46.91411 | Italy | 2011 | CD | present study |  | 20 | 20 | 0 | 0 | 0 | 0 | 20 | 0 |
| Schnalstal | 10.88031 | 46.72386 | Italy | 2011 | TW | present study |  | 24 | 22 | 0 | 2 | 0 | 0 | 22 | 0 |
| Schnann | 10.37955 | 47.15446 | Austria | 2010 | CD | present study |  | 16 | 0 | 16 | 0 | 0 | 0 | 0 | 16 |
| Schrottendorf | 12.67388 | 46.79195 | Austria | 2009 | CD | present study |  | 10 | 0 | 3 | 7 | 0 | 0 | 0 | 3 |
| Scuol-Ftan | 10.26639 | 46.79133 | Switzerland | 2010 | CD | present study |  | 8 | 8 | 0 | 0 | 0 | 0 | 8 | 0 |
| Sent | 10.3495 | 46.81742 | Switzerland | 2015 | HAM, FDN | Nardi et al. (2018) | x | 19 | 19 | 0 | 0 | 0 | 0 | 19 | 0 |
| Serravalle | 11.01438 | 45.84627 | Italy | 2013 | CD | present study |  | 20 | 14 | 3 | 0 | 3 | 0 | 14 | 6 |
| Sigmundskron | 11.30494 | 46.47972 | Italy | 2015 | HAM, FDN | Nardi et al. (2018) | x | 14 | 14 | 0 | 0 | 0 | 0 | 14 | 0 |
| Silz N | 10.91909 | 47.27242 | Austria | 2015 | HAM, FDN | Nardi et al. (2018) | x | 19 | 19 | 0 | 0 | 0 | 0 | 19 | 0 |
| Sinsen | 10.35383 | 47.05372 | Austria | 2015 | HAM, FDN | Nardi et al. (2018) | x | 9 | 0 | 4 | 0 | 0 | 5 | 0 | 9 |
| Soelden | 11.01301 | 46.95997 | Austria | 2015 | HAM, FDN | Nardi et al. (2018) | x | 18 | 0 | 18 | 0 | 0 | 0 | 0 | 18 |
| Soell | 11.243 | 46.35469 | Italy | 2015 | HAM, FDN | Nardi et al. (2018) | x | 19 | 19 | 0 | 0 | 0 | 0 | 19 | 0 |
| Soelles | 10.56327 | 46.6535 | Italy | 2006 | CD | present study |  | 8 | 8 | 0 | 0 | 0 | 0 | 8 | 0 |
| Sonneburg | 11.89358 | 46.78889 | Italy | 2015 | SSt, JAH | Nardi et al. (2018) | x | 10 | 10 | 0 | 0 | 0 | 0 | 10 | 0 |
| Sprechenstein | 11.45618 | 46.88377 | Italy | 2011 | CD | present study |  | 21 | 21 | 0 | 0 | 0 | 0 | 21 | 0 |
| St. Catarina | 13.40105 | 46.50338 | Italy | 2015 | CD | Nardi et al. (2018) | x | 6 | 0 | 0 | 2 | 0 | 4 | 0 | 4 |
| St. Jakob | 11.99053 | 47.00096 | Italy | 2015 | SSt, JAH | Nardi et al. (2018) | x | 20 | 17 | 2 | 1 | 0 | 0 | 17 | 2 |
| St. Justina | 12.58503 | 46.78878 | Austria | 2015 | SSt, JAH | Nardi et al. (2018) | x | 10 | 1 | 9 | 0 | 0 | 0 | 1 | 9 |
| St. Magdalena | 12.23682 | 46.83717 | Italy | 2011 | CD | present study |  | 23 | 0 | 14 | 9 | 0 | 0 | 0 | 14 |
| St. Peter | 12.05884 | 47.02325 | Italy | 2015 | SSt, JAH | Nardi et al. (2018) | x | 12 | 12 | 0 | 0 | 0 | 0 | 12 | 0 |
| St. Sigmund | 11.78566 | 46.81947 | Italy | 2015 | CD | Nardi et al. (2018) | x | 20 | 18 | 2 | 0 | 0 | 0 | 18 | 2 |
| St. Sigmund Sellrain | 11.11333 | 47.20444 | Austria | 2008 | CD | present study |  | 6 | 0 | 6 | 0 | 0 | 0 | 0 | 6 |
| St. Ulrich | 11.63529 | 46.5842 | Italy | 2015 | CD | Nardi et al. (2018) | x | 20 | 0 | 8 | 1 | 11 | 0 | 0 | 19 |
| St. Veit | 12.4221 | 46.92665 | Austria | 2015 | SSt, JAH | Nardi et al. (2018) | x | 11 | 0 | 11 | 0 | 0 | 0 | 0 | 11 |
| Staben | 10.96417 | 46.64806 | Italy | 2009 | JP & RS | present study |  | 5 | 5 | 0 | 0 | 0 | 0 | 5 | 0 |
| Stein | 12.5281 | 47.02602 | Austria | 2015 | SSt, JAH | Nardi et al. (2018) | x | 9 | 0 | 3 | 0 | 2 | 4 | 0 | 9 |
| Stein S | 10.58008 | 46.99653 | Austria | 2010 | CD | present study |  | 14 | 13 | 0 | 1 | 0 | 0 | 13 | 0 |
| Stroeden | 12.3183 | 47.01762 | Austria | 2009 | CD | present study |  | 14 | 0 | 14 | 0 | 0 | 0 | 0 | 14 |
| Strumerhof | 12.51758 | 47.01022 | Austria | 2009 | CD | present study |  | 9 | 0 | 3 | 3 | 3 | 0 | 0 | 6 |
| Tappein | 11.16036 | 46.67886 | Italy | 2010 | CD | present study |  | 15 | 15 | 0 | 0 | 0 | 0 | 15 | 0 |
| Taufers | 11.94939 | 46.9229 | Italy | 2011 | CD | present study |  | 20 | 20 | 0 | 0 | 0 | 0 | 20 | 0 |
| Terlan Mendel | 11.24233 | 46.43081 | Italy | 2015 | HAM, FDN | Nardi et al. (2018) | x | 19 | 19 | 0 | 0 | 0 | 0 | 19 | 0 |
| Terlan N | 11.25383 | 46.5443 | Italy | 2010 | CD & SSc | present study |  | 13 | 12 | 0 | 1 | 0 | 0 | 12 | 0 |
| Thaur | 11.48701 | 47.29489 | Austria | 2015 | HAM, FDN | Nardi et al. (2018) | x | 12 | 12 | 0 | 0 | 0 | 0 | 12 | 0 |
| Thurn Ruine | 12.1135 | 46.76267 | Italy | 2015 | SSt, JAH | Nardi et al. (2018) | x | 12 | 11 | 0 | 1 | 0 | 0 | 11 | 0 |
| Tisens | 11.54825 | 46.57006 | Italy | 2010 | CD & SSc | present study |  | 10 | 0 | 0 | 0 | 0 | 10 | 0 | 10 |
| Toblach | 12.21366 | 46.74275 | Italy | 2015 | SSt, JAH | Nardi et al. (2018) | x | 12 | 11 | 0 | 1 | 0 | 0 | 11 | 0 |
| Toldern | 11.57674 | 47.0916 | Austria | 2015 | HAM, FDN | Nardi et al. (2018) | x | 20 | 0 | 15 | 0 | 4 | 1 | 0 | 20 |
| Trostburg | 11.53644 | 46.58533 | Italy | 2015 | CD | Nardi et al. (2018) | x | 20 | 16 | 0 | 0 | 4 | 0 | 16 | 4 |
| Tschoetscher H | 11.64574 | 46.70514 | Italy | 2015 | CD | Nardi et al. (2018) | x | 20 | 20 | 0 | 0 | 0 | 0 | 20 | 0 |
| Unteralbkus | 12.69247 | 46.87547 | Austria | 2009 | CD | present study |  | 13 | 0 | 7 | 4 | 2 | 0 | 0 | 9 |
| Unterfederaun | 13.8124 | 46.57027 | Austria | 2015 | CD | Nardi et al. (2018) | x | 8 | 0 | 0 | 0 | 5 | 3 | 0 | 8 |
| Unterfennberg | 11.18047 | 46.27308 | Italy | 2015 | HAM, FDN | Nardi et al. (2018) | x | 19 | 0 | 15 | 0 | 4 | 0 | 0 | 19 |
| Unterleibning | 12.63598 | 46.90305 | Austria | 2009 | CD | present study |  | 26 | 0 | 24 | 1 | 0 | 1 | 0 | 25 |
| Unterparggen | 12.36419 | 46.74742 | Italy | 2015 | SSt, JAH | Nardi et al. (2018) | x | 17 | 0 | 6 | 0 | 10 | 1 | 0 | 17 |
| Unterwaldalm | 12.53593 | 47.07657 | Austria | 2009 | CD | present study |  | 18 | 0 | 18 | 0 | 0 | 0 | 0 | 18 |
| Uttenheim | 11.9365 | 46.86932 | Italy | 2011 | CD | present study |  | 20 | 20 | 0 | 0 | 0 | 0 | 20 | 0 |
| Vellau Leiteralm | 11.11092 | 46.70644 | Italy | 2010 | CD | present study |  | 20 | 20 | 0 | 0 | 0 | 0 | 20 | 0 |
| Vellau W | 11.10275 | 46.69303 | Italy | 2010 | CD | present study |  | 14 | 14 | 0 | 0 | 0 | 0 | 14 | 0 |
| Vergein | 12.58432 | 46.79387 | Austria | 2009 | CD | present study |  | 8 | 0 | 8 | 0 | 0 | 0 | 0 | 8 |
| Vernagt | 10.85089 | 46.73864 | Italy | 2015 | HAM, FDN | Nardi et al. (2018) | x | 12 | 10 | 0 | 1 | 1 | 0 | 10 | 1 |
| Vill | 11.40614 | 47.23673 | Austria | 2015 | HAM, FDN | Nardi et al. (2018) | x | 12 | 12 | 0 | 0 | 0 | 0 | 12 | 0 |
| Vinaders | 11.47236 | 47.03329 | Austria | 2015 | HAM, FDN, SSt, JAH | Nardi et al. (2018) | x | 15 | 0 | 10 | 0 | 0 | 5 | 0 | 15 |
| Virgen | 12.45863 | 47.00554 | Austria | 1990 | CD | present study |  | 10 | 0 | 2 | 0 | 8 | 0 | 0 | 10 |
| Voels N | 11.49778 | 46.52592 | Italy | 2006 | CD | present study |  | 10 | 0 | 5 | 0 | 5 | 0 | 0 | 10 |
| Wassermann | 11.82914 | 46.89418 | Italy | 2011 | CD | present study |  | 20 | 20 | 0 | 0 | 0 | 0 | 20 | 0 |
| Welschnofen | 11.54412 | 46.43325 | Italy | 2015 | HAM, FDN | Nardi et al. (2018) | x | 20 | 0 | 4 | 0 | 16 | 0 | 0 | 20 |
| Zabernig | 12.51747 | 47.00618 | Austria | 2009 | CD | present study |  | 16 | 0 | 10 | 0 | 6 | 0 | 0 | 16 |
| Zaunhof | 10.82177 | 47.10001 | Austria | 2010 | CD | present study |  | 17 | 15 | 0 | 2 | 0 | 0 | 15 | 0 |
| Zedlach NW | 12.4899 | 47.00378 | Austria | 2009 | CD | present study |  | 26 | 0 | 25 | 0 | 1 | 0 | 0 | 26 |
| Zernez | 10.10152 | 46.6981 | Switzerland | 2009 | CD | present study |  | 30 | 28 | 0 | 2 | 0 | 0 | 28 | 0 |
| Zerzertal | 10.51728 | 46.75 | Italy | 2015 | HAM, FDN | Nardi et al. (2018) | x | 22 | 12 | 9 | 0 | 1 | 0 | 12 | 10 |
| Zirl Ruine | 11.23942 | 47.27785 | Austria | 2015 | HAM, FDN | Nardi et al. (2018) | x | 20 | 20 | 0 | 0 | 0 | 0 | 20 | 0 |
| Zoesen | 11.77515 | 46.91885 | Italy | 2015 | SSt, JAH | Nardi et al. (2018) | x | 9 | 9 | 0 | 0 | 0 | 0 | 9 | 0 |
| Zwischenwasser | 11.89436 | 46.7234 | Italy | 2011 | CD | present study | Soil subset | 26 | 16 | 9 | 0 | 1 | 0 | 16 | 10 |

**Table A2.** Spearman’s correlation coefficients among ecological variables. Abbreviations: normalised difference vegetation index (NDVI), annual mean temperature (bio01), mean diurnal range (bio02), isothermality (bio03), temperature seasonality (bio04), max temperature of warmest month (bio05), min temperature of coldest month (bio06), temperature annual range (bio07), mean temperature of wettest quarter (bio08), mean temperature of driest quarter (bio09), mean temperature of warmest quarter (bio10), mean temperature of coldest quarter (bio11), annual precipitation (bio12), precipitation of wettest month (bio13), precipitation of driest month (bio14), precipitation seasonality (bio15), precipitation of wettest quarter (bio16), precipitation of driest quarter (bio17), precipitation of warmest quarter (bio18), precipitation of coldest quarter (bio19).

|  | **Elevation** | **Inclination** | **Aspect** | **NDVI** | **bio01** | **bio02** | **bio03** | **bio04** | **bio05** | **bio06** | **bio07** | **bio08** | **bio09** | **bio10** | **bio11** | **bio12** | **bio13** | **bio14** | **bio15** | **bio16** | **bio17** | **bio18** | **bio19** |
| --- | --- | --- | --- | --- | --- | --- | --- | --- | --- | --- | --- | --- | --- | --- | --- | --- | --- | --- | --- | --- | --- | --- | --- |
| Elevation | 1.00 | 0.08 | 0.03 | 0.06 | -0.96 | 0.27 | 0.65 | -0.72 | -0.96 | -0.95 | -0.57 | -0.74 | -0.80 | -0.96 | -0.96 | -0.19 | -0.05 | -0.23 | 0.37 | -0.03 | -0.23 | 0.13 | -0.18 |
| Inclination | 0.08 | 1.00 | 0.02 | 0.10 | -0.09 | 0.00 | 0.13 | -0.17 | -0.10 | -0.07 | -0.14 | 0.05 | -0.01 | -0.09 | -0.07 | -0.13 | -0.14 | -0.05 | -0.01 | -0.14 | -0.05 | -0.13 | -0.05 |
| Aspect | 0.03 | 0.02 | 1.00 | 0.02 | -0.03 | 0.00 | 0.04 | -0.04 | -0.03 | -0.04 | -0.06 | 0.01 | -0.05 | -0.03 | -0.03 | 0.06 | 0.10 | 0.05 | 0.05 | 0.11 | 0.04 | 0.14 | 0.04 |
| NDVI | 0.06 | 0.1 | 0.02 | 1.00 | -0.07 | 0.20 | 0.06 | 0.08 | -0.06 | -0.10 | 0.09 | -0.04 | -0.11 | -0.06 | -0.09 | 0.22 | 0.23 | 0.14 | 0.03 | 0.23 | 0.14 | 0.24 | 0.13 |
| bio01 | -0.96 | -0.09 | -0.03 | -0.07 | 1.00 | -0.35 | -0.72 | 0.73 | 1.00 | 0.99 | 0.57 | 0.77 | 0.84 | 1.00 | 1.00 | 0.15 | 0.00 | 0.19 | -0.36 | -0.03 | 0.19 | -0.20 | 0.13 |
| bio02 | 0.27 | 0.00 | 0.00 | 0.2 | -0.35 | 1.00 | 0.52 | 0.07 | -0.32 | -0.44 | 0.27 | -0.19 | -0.51 | -0.33 | -0.40 | 0.19 | 0.23 | -0.03 | 0.28 | 0.25 | -0.03 | 0.33 | 0.03 |
| bio03 | 0.65 | 0.13 | 0.04 | 0.06 | -0.72 | 0.52 | 1.00 | -0.77 | -0.73 | -0.70 | -0.62 | -0.41 | -0.51 | -0.74 | -0.70 | -0.12 | 0.08 | -0.07 | 0.39 | 0.11 | -0.05 | 0.29 | 0.02 |
| bio04 | -0.72 | -0.17 | -0.04 | 0.08 | 0.73 | 0.07 | -0.77 | 1.00 | 0.76 | 0.64 | 0.95 | 0.43 | 0.38 | 0.75 | 0.67 | 0.36 | 0.16 | 0.17 | -0.31 | 0.14 | 0.16 | -0.03 | 0.12 |
| bio05 | -0.96 | -0.1 | -0.03 | -0.06 | 1.00 | -0.32 | -0.73 | 0.76 | 1.00 | 0.98 | 0.60 | 0.76 | 0.82 | 1.00 | 0.99 | 0.16 | 0.01 | 0.19 | -0.36 | -0.02 | 0.19 | -0.19 | 0.13 |
| bio06 | -0.95 | -0.07 | -0.04 | -0.10 | 0.99 | -0.44 | -0.70 | 0.64 | 0.98 | 1.00 | 0.47 | 0.76 | 0.88 | 0.98 | 1.00 | 0.11 | -0.03 | 0.20 | -0.38 | -0.05 | 0.20 | -0.22 | 0.13 |
| bio07 | -0.57 | -0.14 | -0.06 | 0.09 | 0.57 | 0.27 | -0.62 | 0.95 | 0.60 | 0.47 | 1.00 | 0.31 | 0.23 | 0.59 | 0.50 | 0.40 | 0.21 | 0.18 | -0.27 | 0.20 | 0.17 | 0.05 | 0.15 |
| bio08 | -0.74 | 0.05 | 0.01 | -0.04 | 0.77 | -0.19 | -0.41 | 0.43 | 0.76 | 0.76 | 0.31 | 1.00 | 0.68 | 0.76 | 0.77 | -0.17 | -0.29 | -0.09 | -0.09 | -0.29 | -0.09 | -0.37 | -0.15 |
| bio09 | -0.80 | -0.01 | -0.05 | -0.11 | 0.84 | -0.51 | -0.51 | 0.38 | 0.82 | 0.88 | 0.23 | 0.68 | 1.00 | 0.82 | 0.86 | 0.03 | -0.03 | 0.22 | -0.34 | -0.05 | 0.22 | -0.19 | 0.20 |
| bio10 | -0.96 | -0.09 | -0.03 | -0.06 | 1.00 | -0.33 | -0.74 | 0.75 | 1.00 | 0.98 | 0.59 | 0.76 | 0.82 | 1.00 | 0.99 | 0.16 | 0.00 | 0.19 | -0.36 | -0.02 | 0.19 | -0.20 | 0.12 |
| bio11 | -0.96 | -0.07 | -0.03 | -0.09 | 1.00 | -0.40 | -0.70 | 0.67 | 0.99 | 1.00 | 0.50 | 0.77 | 0.86 | 0.99 | 1.00 | 0.12 | -0.02 | 0.19 | -0.36 | -0.05 | 0.19 | -0.22 | 0.12 |
| bio12 | -0.19 | -0.13 | 0.06 | 0.22 | 0.15 | 0.19 | -0.12 | 0.36 | 0.16 | 0.11 | 0.40 | -0.17 | 0.03 | 0.16 | 0.12 | 1.00 | 0.93 | 0.88 | -0.50 | 0.92 | 0.88 | 0.83 | 0.86 |
| bio13 | -0.05 | -0.14 | 0.10 | 0.23 | 0.00 | 0.23 | 0.08 | 0.16 | 0.01 | -0.03 | 0.21 | -0.29 | -0.03 | 0.00 | -0.02 | 0.93 | 1.00 | 0.83 | -0.28 | 1.00 | 0.83 | 0.94 | 0.84 |
| bio14 | -0.23 | -0.05 | 0.05 | 0.14 | 0.19 | -0.03 | -0.07 | 0.17 | 0.19 | 0.20 | 0.18 | -0.09 | 0.22 | 0.19 | 0.19 | 0.88 | 0.83 | 1.00 | -0.68 | 0.82 | 1.00 | 0.74 | 0.98 |
| bio15 | 0.37 | -0.01 | 0.05 | 0.03 | -0.36 | 0.28 | 0.39 | -0.31 | -0.36 | -0.38 | -0.27 | -0.09 | -0.34 | -0.36 | -0.36 | -0.50 | -0.28 | -0.68 | 1.00 | -0.25 | -0.68 | -0.13 | -0.66 |
| bio16 | -0.03 | -0.14 | 0.11 | 0.23 | -0.03 | 0.25 | 0.11 | 0.14 | -0.02 | -0.05 | 0.20 | -0.29 | -0.05 | -0.02 | -0.05 | 0.92 | 1.00 | 0.82 | -0.25 | 1.00 | 0.83 | 0.96 | 0.83 |
| bio17 | -0.23 | -0.05 | 0.04 | 0.14 | 0.19 | -0.03 | -0.05 | 0.16 | 0.19 | 0.20 | 0.17 | -0.09 | 0.22 | 0.19 | 0.19 | 0.88 | 0.83 | 1.00 | -0.68 | 0.83 | 1.00 | 0.74 | 0.98 |
| bio18 | 0.13 | -0.13 | 0.14 | 0.24 | -0.20 | 0.33 | 0.29 | -0.03 | -0.19 | -0.22 | 0.05 | -0.37 | -0.19 | -0.20 | -0.22 | 0.83 | 0.94 | 0.74 | -0.13 | 0.96 | 0.74 | 1.00 | 0.75 |
| bio19 | -0.18 | -0.05 | 0.04 | 0.13 | 0.13 | 0.03 | 0.02 | 0.12 | 0.13 | 0.13 | 0.15 | -0.15 | 0.20 | 0.12 | 0.12 | 0.86 | 0.84 | 0.98 | -0.66 | 0.83 | 0.98 | 0.75 | 1.00 |

**Table A3.** Spearman’s correlation coefficients among soil variables. Abbreviations: C_org_ organic carbon; N_tot_ total nitrogen; CEC cation exchange capacity; BS base saturation.

|  | pH | CO_3_^2-^ | C_org_ | N_tot_ | C/N | K^+^ | Na^+^ | Ca^2+^ | Mg^2+^ | Mn^3+^ | Al^3+^ | Fe^3+^ | H^+^ | CEC | BS |
| --- | --- | --- | --- | --- | --- | --- | --- | --- | --- | --- | --- | --- | --- | --- | --- |
| pH | 1.00 | 0.83 | 0.18 | 0.11 | 0.15 | -0.32 | -0.17 | 0.54 | 0.24 | -0.93 | -0.75 | -0.70 | -0.65 | 0.51 | 0.79 |
| CO_3_^2-^ | 0.83 | 1.00 | 0.29 | 0.18 | 0.22 | -0.44 | -0.17 | 0.36 | 0.21 | -0.77 | -0.45 | -0.53 | -0.33 | 0.35 | 0.51 |
| C_org_ | 0.18 | 0.29 | 1.00 | 0.95 | -0.01 | 0.12 | 0.29 | 0.72 | 0.56 | -0.08 | 0.01 | 0.13 | -0.03 | 0.77 | 0.20 |
| N_tot_ | 0.11 | 0.18 | 0.95 | 1.00 | -0.28 | 0.15 | 0.33 | 0.72 | 0.55 | 0.00 | 0.03 | 0.15 | -0.02 | 0.77 | 0.14 |
| C/N | 0.15 | 0.22 | -0.01 | -0.28 | 1.00 | -0.02 | -0.17 | -0.15 | -0.02 | -0.19 | -0.02 | -0.04 | 0.00 | -0.15 | 0.09 |
| K^+^ | -0.32 | -0.44 | 0.12 | 0.15 | -0.02 | 1.00 | 0.23 | 0.02 | 0.14 | 0.28 | 0.07 | 0.07 | 0.09 | 0.06 | -0.09 |
| Na^+^ | -0.17 | -0.17 | 0.29 | 0.33 | -0.17 | 0.23 | 1.00 | 0.17 | 0.21 | 0.15 | 0.07 | 0.12 | 0.20 | 0.21 | -0.12 |
| Ca^2+^ | 0.54 | 0.36 | 0.72 | 0.72 | -0.15 | 0.02 | 0.17 | 1.00 | 0.48 | -0.41 | -0.49 | -0.23 | -0.51 | 0.99 | 0.60 |
| Mg^2+^ | 0.24 | 0.21 | 0.56 | 0.55 | -0.02 | 0.14 | 0.21 | 0.48 | 1.00 | -0.19 | -0.15 | -0.12 | -0.30 | 0.59 | 0.35 |
| Mn^3+^ | -0.93 | -0.77 | -0.08 | 0.00 | -0.19 | 0.28 | 0.15 | -0.41 | -0.19 | 1.00 | 0.69 | 0.67 | 0.58 | -0.38 | -0.81 |
| Al^3+^ | -0.75 | -0.45 | 0.01 | 0.03 | -0.02 | 0.07 | 0.07 | -0.49 | -0.15 | 0.69 | 1.00 | 0.75 | 0.70 | -0.45 | -0.75 |
| Fe^3+^ | -0.70 | -0.53 | 0.13 | 0.15 | -0.04 | 0.07 | 0.12 | -0.23 | -0.12 | 0.67 | 0.75 | 1.00 | 0.56 | -0.21 | -0.59 |
| H^+^ | -0.65 | -0.33 | -0.03 | -0.02 | 0.00 | 0.09 | 0.20 | -0.51 | -0.30 | 0.58 | 0.70 | 0.56 | 1.00 | -0.48 | -0.77 |
| CEC | 0.51 | 0.35 | 0.77 | 0.77 | -0.15 | 0.06 | 0.21 | 0.99 | 0.59 | -0.38 | -0.45 | -0.21 | -0.48 | 1.00 | 0.58 |
| BS | 0.79 | 0.51 | 0.20 | 0.14 | 0.09 | -0.09 | -0.12 | 0.60 | 0.35 | -0.81 | -0.75 | -0.59 | -0.77 | 0.58 | 1.00 |

**Table A4.** Pairwise comparison of soil variables among sexual and apomictic cytotypes of *Potentilla puberula*. Coefficients of regression ± standard errors are given. Positive and negative coefficients indicate higher and lower values for the cytotypes given first in the column headings, respectively. No significant difference was found for any comparison. Abbreviations: N_tot_ total nitrogen.

|  | Sexuals vs Apomicts | | | Apomicts vs Apomicts | | |
| --- | --- | --- | --- | --- | --- | --- |
| Variable | **Tetra- vs Pentaploids** | **Tetra- vs Heptaploids** | **Tetra- vs Octoploids** | **Penta- vs Heptaploids** | **Penta- vs Octoploids** | **Hepta- vs Octoploids** |
| CO_3_^2-^ | 0.29 ± 0.20 | 0.15 ± 0.22 | -0.04 ± 0.24 | -0.16 ± 0.21 | -0.33 ± 0.24 | -0.21 ± 0.27 |
| N_tot_ | 0.06 ± 0.18 | -0.26 ± 0.20 | -0.02 ± 0.25 | -0.30 ± 0.20 | -0.09 ± 0.26 | 0.28 ± 0.32 |
| C/N | 0.12 ± 0.19 | 0.01 ± 0.21 | 0.00 ± 0.25 | -0.10 ± 0.22 | -0.10 ± 0.25 | -0.01 ± 0.25 |
| K^+^ | 0.09 ± 0.18 | -0.13 ± 0.19 | 0.28 ± 0.30 | -0.19 ± 0.20 | 0.14 ± 0.27 | 0.32 ± 0.31 |
| Na^+^ | -0.05 ± 0.18 | -0.13 ± 0.20 | 0.20 ± 0.31 | -0.09 ± 0.20 | 0.29 ± 0.30 | 0.42 ± 0.33 |
| Ca^2+^ | 0.07 ± 0.18 | -0.24 ± 0.20 | -0.03 ± 0.25 | -0.34 ± 0.20 | -0.11 ± 0.25 | 0.28 ± 0.31 |
| Mg^2+^ | -0.12 ± 0.17 | -0.33 ± 0.20 | -0.25 ± 0.23 | -0.21 ± 0.20 | -0.14 ± 0.26 | 0.12 ± 0.30 |
| Al^3+^ | -0.01 ± 0.18 | 0.91 ± 0.67 | 0.39 ± 0.44 | 1.02 ± 0.77 | 0.42 ± 0.49 | -0.19 ± 0.31 |
| Fe^3+^ | -0.14 ± 0.19 | 0.24 ± 0.23 | 0.03 ± 0.25 | 0.52 ± 0.35 | 0.20 ± 0.33 | -0.23 ± 0.28 |
| H^+^ | 0.08 ± 0.18 | 0.73 ± 0.47 | 0.38 ± 0.39 | 0.67 ± 0.44 | 0.30 ± 0.35 | -0.17 ± 0.30 |

**Table A5.** Survey of published studies on ecological parthenogenesis of sexual-apomictic taxa. “Ploidy of sexuals” and “apomicts” determined within the cited study are given (in brackets, the ploidy of known but not assessed cytotypes is reported). We distinguish between an auto- and allopolyploid “origin of apomicts”, and the question mark indicates a possible allopolyploid origin of apomicts. “Taxonomic relationship” indicates whether sexual-apomictic contrasts occur at the species (intraspecific) or generic level (interspecific). The geographic distribution of reproductive modes relative to each other is classified as sympatry, parapatry, allopatry, and “parasympatry”, the latter here defined as the condition in which apomicts have a wider geographic distribution which totally includes that of the sexuals. “Ecological differentiation” between sexual and apomict cytotypes (“yes” or “no”) and whether an “immediate role of ecology as a driver of the observed distribution pattern” can be told apart from effects related to differential migration abilities of reproductive modes (“yes”, “no” or “not applicable”, in case of no ecological differentiation) are also given. We further conclude on whether the ecological distribution pattern can be attributed to effects of reproductive mode, ploidy level, or hybridisation. The reasons for “uncertainty” are provided according to the following scheme: evolutionary origin of apomictic cytotypes unknown (A), ploidy level was not assessed within the study (B), evolutionary divergence or hybridity (C), reproductive mode was not assessed within the study (D), sexual contrast missing (E), existence of only one cytotype linked to one reproductive mode (F), relationship among apomictic ploidy levels not studied (G). The “main result” of the study is also reported.

| **Taxon** | **Ploidy of sexuals** | **Ploidy of apomicts** | **Origin of apomicts** | **Taxonomic relationship of sexuals and apomicts** | **Geographical relationship of sampled sexual and apomictic populations** | **Ecological differentiation** | **Can an immediate role of ecology as a driver of the observed differentiation be told apart from spatial effects?** | **Ecological differentiation pattern attributable to ploidy level, reproductive mode or other factors?** | **Main result** | **Reference** |
| --- | --- | --- | --- | --- | --- | --- | --- | --- | --- | --- |
| *Antennaria parlinii* Fernald | 6x | 6x | ? | Intraspecific | Common garden (individuals originally sampled from a sympatric area) | Yes | Yes | Uncertainty: A | Apomicts live in more disturbed habitats and sexuals in open wooded sites.  Apomicts increase reproductive allocation when more light and nutrients are available, while sexuals do not. | Michaels and Bazzaz (1989) |
| *Antennaria rosea* Greene and sexual progenitors^[[1]](#footnote-1)^ | (2x, 3x, 4x, 6x, 7x, 8x, 10x)^[[2]](#footnote-2)^ | (3x)  (4x)  (5x)^[[3]](#footnote-3)^ | Allopolyploid | Interspecific | Parasympatry | Yes | No | Uncertainty: B, C, D | Apomicts occupy intermediate or similar niches compared to parental sexual species | Bayer et al. (1991) |
| *Arnica cordifolia* Hook. |  | 3x  4x | Autopolyploid^[[4]](#footnote-4)^ | Intraspecific | Mixed populations | No | Not applicable | Uncertainty:  E | No habitat differentiation among apomictic cytotypes. | Kao (2008); Kao and Parker (2010) |
| *Boechera* Á. Löve & D. Löve | 2x  3x | 2x  3x | Species- and ploidy-specific^[[5]](#footnote-5)^ | Inter- and intraspecific | Mostly sympatry  Correction for geographical distances | Yes | Yes | Uncertainty: C | Niche differentiation between sexual cytotypes but not between apomictic cytotypes.  Niche differentiation between sexual and apomictic diploids. | Mau et al. (2015) |
| *Crataegus* subgen. *Sanguineae* Ufimov | 2x | 3x  4x  5x | Auto- and allopolyploid | Inter- and intraspecific | Allopatry | Yes | No | Uncertainty: C | Allopolyploids inhabit cooler and more seasonally variable habitats than sexuals and autotriploids, and their niches are intermediate to progenitors.  Niche broadening is associated with allopolyploidy. | Coughlan et al. (2017) |
| *Erigeron strigosus* Muhl. ex Willd. | (2x) | (3x)  (4x)  (6x)^[[6]](#footnote-6)^ | ? | Intraspecific | Parasympatry | Yes | No | Uncertainty: A, B | Apomicts have wider niches in terms of precipitation and soil properties. | Noyes et al. (2006) |
| *Hieracium alpinum* L. | 2x | 3x | Autopolyploid | Intraspecific | Allopatry | No | Not applicable | Uncertainty: F | No differences in eco-physiological plant traits among cytotypes. | Hartmann et al. (2018) |
|  |  |  |  |  |  | No | Not applicable | Uncertainty: F | No differences among cytotypes in biotic interactions (Red Queen). | Hartmann et al. (2017) |
| *Limonium* Mill. | 2x | 3x (?)  4x^[[7]](#footnote-7)^ | ? | Interspecific | Sympatry | Yes | Yes | Uncertainty: A, D | Apomicts show broader niches than sexuals, which inhabit drier and warmer sites. | Caperta et al. (2017) |
| *Paspalum intermedium* Munro ex Morong | 2x | 4x | Autopolyploid | Intraspecific | Parapatry | Yes | No | Uncertainty: F | Apomicts inhabit colder and drier habitats than sexuals and have broader ecological niches. | Karunarathne et al. (2018) |
| *Pilosella officinarum* Vaill. | 6x | 5x  6x | ? | Intraspecific | Allopatry | Yes^[[8]](#footnote-8)^ | No | Uncertainty: A, D | Pentaploids occur at higher elevation than hexaploids. | Mráz et al. (2008) |
| *Poa cusickii* Vasey subsp. *pallida* Soreng  *P. fendleriana* (Steud.) Vasey subsp. *fendleriana*  and subsp. *longiligula* (Scribn. & T.A.Williams) Soreng | (8x)^[[9]](#footnote-9)^ | (8x)^9^ | Likely autopolyploid^[[10]](#footnote-10)^ | Intraspecific | Parasympatry | Yes | No^[[11]](#footnote-11)^ | Uncertainty: B | Apomicts have broader ecological niches than sexuals and are more successful in cooler habitats with short frost-free season | Soreng (2000) |
| *Poa nervosa* (Hook.) Vasey and *P. wheeleri* Vasey | (4x)^9^ | (8-13x)^9^ | Allopolyploid^[[12]](#footnote-12)^ | Interspecific | Allopatry | Yes | No | Uncertainty: B, C | Apomicts have broader ecological niches than sexuals and are more successful in cooler habitats with short frost-free season | Soreng (2000) |
| *Potentilla puberula* Kra[š](https://www.tandfonline.com/author/Dobe%C5%A1%2C+Christoph)an | 4x | 5x, 7x, 8x | Autopolyploid^[[13]](#footnote-13)^ | Intraspecific | Sympatry | Yes | Yes | Reproductive mode | Apomicts are more frequent in human-disturbed and wetter habitats, while sexuals occur in steeper and more south-oriented sites. | Present study |
| *Ranunculus auricomus* complex | 2x  4x (autopolyploid) | 3-8x | Allopolyploid^[[14]](#footnote-14)^ | Mostly interspecific | Parasympatry | Yes | No | Uncertainty: C, D, G | Apomicts inhabit cooler and drier sites. | Paule et al. (2018) |
| *Ranunculus carpaticola* Soó | 2x | 6x | Allopolyploid^[[15]](#footnote-15)^ | Intraspecific^[[16]](#footnote-16)^ | Allopatric | Yes | No | Uncertainty: C, F | Apomicts inhabit a higher variety of habitats than sexuals. | Paun et al. (2006a) |
| *Ranunculus kuepferi* Greuter & Burdet | 2x | 4x | Autopolyploid^[[17]](#footnote-17)^ | Intraspecific | Parapatry | Yes | No | Uncertainty: F^[[18]](#footnote-18)^ | 4x apomicts occur at higher elevation and under the related climatic factors. | Schinkel et al. (2016) |
|  |  |  |  |  | Sympatry | Partial | Yes | Uncertainty: F | incomplete niche divergence between apomicts and sexuals. | Kirchheimer et al. (2016) |
|  |  |  |  |  | Allopatry | Yes | No | Uncertainty: F | Niche shift between apomicts and sexuals. | Kirchheimer et al. (2016) |
| *Taraxacum* sect. *Ruderalia* Kirschner, H.Øllg. & Štěpánek | 2x | 3x | Autopolyploid^[[19]](#footnote-19)^ | Intraspecific | Mixed populations | Yes | Yes | Uncertainty: F | Apomicts inhabit all microhabitats, while sexuals live in warmer and more southern slopes. | Verduijn et al. (2004) |
|  |  |  |  |  | Mixed populations | Yes | Yes | Uncertainty: F | Apomicts are associated with human-disturbed areas (at lower elevation), sexuals with more stable sites at higher elevation. | Meirmans et al. (1999) |

**References**

Bayer, R.J., Chandler, G.T., 2007. Evolution of polyploid agamic complexes: a case study using the *Catipes* group of *Antennaria*, including the *A. rosea* complex (Asteraceae: Gnaphalieae), in: Hörandl, E., Grossniklaus, U., van Dijk, P.J., Sharbel, T.F. (Eds.), Apomixis: Evolution, Mechanisms and Perspectives. A. R. G. Gantner Verlag, Rugell, Ruggell, Liechtenstein, pp. 317–336.

Bayer, R.J., Purdy, B.G., Lebedyk, D.G., 1991. Niche differentiation among eight sexual species of *Antennaria* Gaertner (Asteraceae: Inuleae) and *A. rosea*, their allopolyploid derivative. Evol. Trends Plants 5, 109–123.

Bayer, R.J., Stebbins, G.L., 1987. Chromosome numbers, patterns of distribution, and apomixis in *Antennaria* (Asteraceae: Inuleae). Syst. Bot. 12, 305–319. https://doi.org/10.2307/2419326

Caperta, A.D., Castro, S., Loureiro, J., Róis, A.S., Conceição, S., Costa, J., Rhazi, L., Espírito Santo, D., Arsénio, P., 2017. Biogeographical, ecological and ploidy variation in related asexual and sexual *Limonium* taxa ( *Plumbaginaceae* ). Bot. J. Linn. Soc. 183, 75–93. https://doi.org/10.1111/boj.12498

Cosendai, A.-C., Wagner, J., Ladinig, U., Rosche, C., Hörandl, E., 2013. Geographical parthenogenesis and population genetic structure in the alpine species *Ranunculus kuepferi* (Ranunculaceae). Heredity (Edinb). 110, 560–569. https://doi.org/10.1038/hdy.2013.1

Coughlan, J.M., Han, S., Stefanović, S., Dickinson, T.A., 2017. Widespread generalist clones are associated with range and niche expansion in allopolyploids of Pacific Northwest Hawthorns (*Crataegus* L.). Mol. Ecol. 26, 5484–5499. https://doi.org/10.1111/mec.14331

Dobeš, C., Sharbel, T.F., Koch, M., 2007. Towards understanding the dynamics of hybridization and apomixis in the evolution of the genus *Boechera* (Brassicaceae). Syst. Biodivers. 5, 321–331. https://doi.org/10.1017/S1477200007002423

Hartmann, M., Jandová, K., Chrtek, J., Štefánek, M., Mráz, P., 2018. Effects of latitudinal and elevational gradients exceed the effects of between-cytotype differences in eco-physiological leaf traits in diploid and triploid *Hieracium alpinum*. Alp. Bot. 128, 133–147. https://doi.org/10.1007/s00035-018-0210-9

Hartmann, M., Štefánek, M., Zdvořák, P., Heřman, P., Chrtek, J., Mráz, P., 2017. The Red Queen hypothesis and geographical parthenogenesis in the alpine hawkweed *Hieracium alpinum* (Asteraceae). Biol. J. Linn. Soc. 122, 681–696. https://doi.org/10.1093/biolinnean/blx105

Hörandl, E., Greilhuber, J., Klímová, K., Paun, O., Temsch, E., Emadzade, K., 2009. Reticulate evolution and taxonomic concepts in the *Ranunculus auricomus* complex (Ranunculaceae): insights from analysis of morphological, karyological and molecular data. Taxon 58, 1194–1216.

Kao, R.H., 2008. Origins and widespread distribution of co-existing polyploids in *Arnica cordifolia* (Asteraceae). Ann. Bot. 101, 145–52. https://doi.org/10.1093/aob/mcm271

Kao, R.H., Parker, I.M., 2010. Coexisting cytotypes of *Arnica cordifolia*: morphological differentiation and local‐scale distribution. Int. J. Plant Sci. 171, 81–89. https://doi.org/10.1086/647924

Karunarathne, P., Schedler, M., Martínez, E.J., Honfi, A.I., Novichkova, A., Hojsgaard, D., 2018. Intraspecific ecological niche divergence and reproductive shifts foster cytotype displacement and provide ecological opportunity to polyploids. Ann. Bot. 121, 1183–1196. https://doi.org/10.1093/aob/mcy004

Kirchheimer, B., Schinkel, C.C.F., Dellinger, A.S., Klatt, S., Moser, D., Winkler, M., Lenoir, J., Caccianiga, M., Guisan, A., Nieto-Lugilde, D., Svenning, J.-C., Thuiller, W., Vittoz, P., Willner, W., Zimmermann, N.E., Hörandl, E., Dullinger, S., 2016. A matter of scale: apparent niche differentiation of diploid and tetraploid plants may depend on extent and grain of analysis. J. Biogeogr. 43, 716–726. https://doi.org/10.1111/jbi.12663

Lovell, J.T., Aliyu, O.M., Mau, M., Schranz, M.E., Koch, M.A., Kiefer, C., Song, B.-H., Mitchell-Olds, T., Sharbel, T.F., 2013. On the origin and evolution of apomixis in *Boechera*. Plant Reprod. 26, 309–315. https://doi.org/10.1007/s00497-013-0218-7

Mau, M., Lovell, J.T., Corral, J.M., Kiefer, C., Koch, M.A., Aliyu, O.M., Sharbel, T.F., 2015. Hybrid apomicts trapped in the ecological niches of their sexual ancestors. PNAS 112, E2357–E2365. https://doi.org/10.1073/pnas.1423447112

Meirmans, P.G., Calame, F.G., Bretagnolle, F., Felber, F., den Nijs, J.C.M., 1999. Anthropogenic disturbance and habitat differentiation between sexual diploid and apomictic triploid *Taraxacum* sect. *Ruderalia*. Folia Geobot. 34, 451–469. https://doi.org/10.1007/BF02914922

Michaels, H.J., Bazzaz, F.A., 1989. Individual and population responses of sexual and apomictic plants to environmental gradients. Am. Nat. 134, 190–207. https://doi.org/10.1086/284975

Mráz, P., Šingliarová, B., Urfus, T., Krahulec, F., 2008. Cytogeography of *Pilosella officinarum* (Compositae): altitudinal and longitudinal differences in ploidy level distribution in the Czech Republic and Slovakia and the general pattern in Europe. Ann. Bot. 101, 59–71. https://doi.org/10.1093/aob/mcm282

Nardi, F.D., Dobeš, C., Müller, D., Grasegger, T., Myllynen, T., Alonso-Marcos, H., Tribsch, A., 2018. Sexual intraspecific recombination but not de novo origin governs the genesis of new apomictic genotypes in Potentilla puberula (Rosaceae). Taxon 67, 1108–1131. https://doi.org/ 10.12705/676.8.

Noyes, R.D., Gerling, H., Vandervoort, C., 2006. Sexual and apomictic prairie fleabane (*Erigeron strigosus*) in Texas: geographic analysis and a new combination (*Erigeron strigosus* var. *traversii*, Asteraceae). SIDA, Contrib. to Bot. 22, 265–276.

Paule, J., Dunkel, F.G., Schmidt, M., Gregor, T., 2018. Climatic differentiation in polyploid apomictic *Ranunculus auricomus* complex in Europe. BMC Ecol. 18, 16. https://doi.org/10.1186/s12898-018-0172-1

Paun, O., Greilhuber, J., Temsch, E.M., Hörandl, E., 2006a. Patterns, sources and ecological implications of clonal diversity in apomictic *Ranunculus carpaticola* (*Ranunculus auricomus* complex, Ranunculaceae). Mol. Ecol. 15, 897–910. https://doi.org/10.1111/j.1365-294X.2006.02800.x

Paun, O., Stuessy, T.F., Hörandl, E., 2006b. The role of hybridization, polyploidization and glaciation in the origin and evolution of the apomictic *Ranunculus cassubicus* complex. New Phytol. 171, 223–236. https://doi.org/10.1111/j.1469-8137.2006.01738.x

Schinkel, C.C.F., Kirchheimer, B., Dellinger, A.S., Klatt, S., Winkler, M., Dullinger, S., Hörandl, E., 2016. Correlations of polyploidy and apomixis with elevation and associated environmental gradients in an alpine plant. AoB Plants 8, plw064. https://doi.org/10.1093/aobpla/plw064

Soreng, R.J., 2007. *Poa* L., in: Barkworth, M.E., Capels, K.M., Long, S., Anderton, L.K., Piep, M.B. (Eds.), Magnoliophyta: Commelinidae (in Part): Poaceae, Part 1. Flora of North America North of Mexico, 24. Oxford University Press, New York, Oxford, pp. 486–601.

Soreng, R.J., 2000. Apomixis and amphimixis comparative biogeography: a study in *Poa* (Poaceae), in: Jacobs, S.W.L., Everett, J. (Eds.), Grasses: Systematics and Evolution. CSIRO, Collingwood, Australia, pp. 294–306.

Soreng, R.J., 1991. Systematics of the “Epiles” group of *Poa* (Poaceae). Syst. Bot. 16, 507–528. https://doi.org/10.2307/2419340

Soreng, R.J., 1986. Distribution and evolutionary significance of apomixis in diclinous *Poa* of western North America. New Mexico State University, Las Cruces.

Verduijn, M.H., Van Dijk, P.J., Van Damme, J.M.M., 2004. Distribution, phenology and demography of sympatric sexual and asexual dandelions (*Taraxacum officinale s.l.*): geographic parthenogenesis on a small scale. Biol. J. Linn. Soc. 82, 205–218. https://doi.org/10.1111/j.1095-8312.2004.00325.x

**Appendix S2: Soil analyses**

**Material and Methods**

We sampled soil of the upper 10 cm (including the humus layer and the first mineral horizon) from 121 populations (Appendix 1) to analyse the following soil parameters: soil acidity (pH), carbonate content (CO_3_^2-^), organic carbon (C_org_) and total nitrogen content (N_tot_), cation content and cation exchange capacity (CEC). Soil samples varied from approximately 200 g to 1050 g. We determined values for pH with 5 ml spoon of soil in 25 ml CaCl_2_ 0.01 M solution, stirred with a glass rod and incubated for at least two hours at room temperature for pH meter measurement (pH meter 713, Metrohm, Switzerland).

For measuring CO_3_^2-^ we filled the Schleiber Unit with sealing fluid (deionized water acidified with H_2_SO_4_) up to the zero point. We added diluted HCl in deionized water to 2g of soil and measured the released CO_2_ displacing the sealing fluid. We compared the obtained values to 6–16 control samples of different Austrian soils: values below or above 6 indicate absence or presence of CO_3_^2-^, respectively. We calculated C_org_ and N_tot_ with the elemental analyser (TruMac CN, LECO, United States) by dry combustion of 400 mg of soil burned in a pure oxygen atmosphere at 1,250 °C, converting the C in CO_2_ and the N in N_2_ and NO_x_, NO_x_ gases are reduced to N_2_ through a copper catalyst. We determined C_org_ as the difference of total carbon and CO_3_^2-^.

Subsequently, we estimated the CEC using an Inductively Coupled Plasma Optical Emission Spectrometry Optima 8300 (ICP-OES, Perkin Elmer, United States). We kept 5 g of soil overnight in 100 ml BaCl_2_ 0.1 M to equilibrate the samples and we shook them for two hours and filtrating them afterwards. In the filtrate we determined the H^+^-ion by pH meter measurement and all the other cations (K^+^, Na^+^, Ca^2+^, Mg^2+^, Fe^3+^, Al ^3+^, Mn^3+^) by ICP-OES. For soils with CO_3_^2-^ content, as the Ca^2+^-ion and Mg^2+^-ion were also dissolved, 100 ml HCl 0.2 M were added to replace the Ba^2+^ ions and make a mathematical correction for the dissolved cations. We used four mix solutions containing all the cations (K^+^, Na^+^, Ca^2+^, Mg^2+^, Fe^3+^, Al^3+^, Mn^3+^) as calibration standards for ICP-OES measurements. We introduced the samples into inductively coupled argon plasma via a pneumatic atomisation system and at 9,726.85 °C the elements in the solution were excited to light emission. The emitted light was split via an echelle-based polychromator into element-specific wavelengths and detected for simultaneous analysis. The CEC is the sum of all cations: Fe^3+^, Al^3+^, Mn^3+^ and H^+^ have an acid effect, whereas K^+^, Na^+^, Ca^2+^ and Mg^2+^ have a basic acting. Therefore, we calculated the base saturation by dividing the sum of K^+^+ Na^+^+ Ca^2+^ + Mg^2+^ through the CEC and multiplied per 100, which gave us the result in percentages.

We removed highly inter-correlating soil variables (i.e., |Spearman correlation coefficient| > 0.75) from the analyses (Table A3). The then regressed the probability of occurrence of a cytotype (i.e., 0 or 1 in single-cytotype populations and 0.5 in mixed populations) onto the remaining soil variables (CO_3_^2-^, N_tot_, C/N, K^+^, Na^+^, Ca^2+^, Mg^2+^, Al^3+^, Fe^3+^ and H^+^) by means of logistic regressions as described in the main text for the other ecological variables.

**Results**

We found no significant differences among cytotypes for any of the studied soil variable (Table A4).

1. *Antennaria parlinii* is an exclusively apomictic allopolyploid which was compared to its sexual progenitor species: *A. aromatica* Evert, *A. corymbosa* Nelson, *A. marginata* Greene, *A. media* Greene, *A. microphylla* Rydb., *A. racemosa* Hook., *A. rosulata* Rydb. and *A. umbrinella* Rydb. [↑](#footnote-ref-1)
2. Bayer and Stebbins (1987). [↑](#footnote-ref-2)
3. Bayer and Chandler (2007). [↑](#footnote-ref-3)
4. Kao (2008). [↑](#footnote-ref-4)
5. Hybridisation is known to play an important role in the diversification of the genus (Dobeš et al., 2007). In three species, apomictic diploids have been proven to be conspecific with sexuals, while apomictic triploids to derive by hybridisation (Lovell et al., 2013). [↑](#footnote-ref-5)
6. Apomictic individuals were determined as such when they produced a high proportion of aborted pollen grains, but ploidy was not directly addressed. [↑](#footnote-ref-6)
7. Three species complexes were sampled in this study, each representing a single ploidy level. The reproductive mode of the triploids, *L. algarvense* Erben, is unknown (Caperta et al., 2017). [↑](#footnote-ref-7)
8. In this case, a differentiation between pentaploids and hexaploids was made, without taking into consideration reproductive mode. [↑](#footnote-ref-8)
9. Ploidy level was not assessed within the study but was assumed on the basis of known chromosome counts (Soreng, 1991, 1986). Since these *Poa* species are dioecious, reproductive mode was estimated at the population level by the male: female plants ratios. [↑](#footnote-ref-9)
10. R. J. Soreng, personal communication. [↑](#footnote-ref-10)
11. Actually, (Soreng, 2000) distinguished between pistillate plants from exclusively apomictic regions and pistillate plants from regions where male plants occurred as well. However, no distinction in the reproductive mode of the sympatric pistillate plants was made. [↑](#footnote-ref-11)
12. Soreng (2007). [↑](#footnote-ref-12)
13. Nardi et al. (2018). [↑](#footnote-ref-13)
14. Hörandl et al. (2009). [↑](#footnote-ref-14)
15. Paun et al. (2006b). [↑](#footnote-ref-15)
16. The apomictic cytotype is currently treated as the hybrid form *R. carpaticola* × *cassubicifolius* (Hörandl et al., 2009). [↑](#footnote-ref-16)
17. Cosendai et al. (2013). [↑](#footnote-ref-17)
18. Schinkel et al. (2016) actually found rare apomictic diploids within the sexual geographical and ecological range. We are not considering them here, as their numbers were too low to draw conclusions on the role of ploidy and reproductive mode in determining the ecological differentiation. [↑](#footnote-ref-18)
19. P. Meirmans (personal communication). [↑](#footnote-ref-19)
